# Genome-wide prediction of pathogenic gain- and loss-of-function variants from ensemble learning of a diverse feature set

**DOI:** 10.1186/s13073-023-01261-9

**Published:** 2023-11-30

**Authors:** David Stein, Meltem Ece Kars, Yiming Wu, Çiğdem Sevim Bayrak, Peter D. Stenson, David N. Cooper, Avner Schlessinger, Yuval Itan

**Affiliations:** 1 Department of Pharmacological Sciences, Icahn School of Medicine at Mount Sinai, New York, NY 10029 USA; 2 The Charles Bronfman Institute for Personalized Medicine, Icahn School of Medicine at Mount Sinai, New York, NY 10029 USA; 3 Department of Genetics and Genomic Sciences, Icahn School of Medicine at Mount Sinai, New York, NY 10029 USA; 4 College of Life Science, China West Normal University, Nan Chong, Si Chuan 637009 China; 5 Institute of Medical Genetics, School of Medicine, Cardiff University, Cardiff, CF14 4XN UK; 6 Department of Artificial Intelligence and Human Health, Icahn School of Medicine at Mount Sinai, New York, NY 10029 USA

**Keywords:** Gain-of-function, Loss-of-function, Protein function, Variant functional impact, Pathogenicity prediction, Precision medicine, Genomic medicine, Phenome-wide association studies, Natural language processing, Machine learning

## Abstract

Gain-of-function (GOF) variants give rise to increased/novel protein functions whereas loss-of-function (LOF) variants lead to diminished protein function. Experimental approaches for identifying GOF and LOF are generally slow and costly, whilst available computational methods have not been optimized to discriminate between GOF and LOF variants. We have developed LoGoFunc, a machine learning method for predicting pathogenic GOF, pathogenic LOF, and neutral genetic variants, trained on a broad range of gene-, protein-, and variant-level features describing diverse biological characteristics. LoGoFunc outperforms other tools trained solely to predict pathogenicity for identifying pathogenic GOF and LOF variants and is available at https://itanlab.shinyapps.io/goflof/.

**Supplementary Information:**

The online version contains supplementary material available at 10.1186/s13073-023-01261-9.

## Background

Genetic variations exert diverse functional effects on gene products and can impact protein stability, interactions with binding partners, and catalytic activity, among many other properties [1]. It is essential to investigate the functional consequences of genetic variations to understand their impact on the diverse array of observed human disease phenotypes. In particular, the functional consequences of genetic variations include two broad categories: gain-of-function (GOF) variants, characterized by enhanced or novel protein activity, and loss-of-function (LOF) variants which result in partial or complete knockdown of protein activity. GOF and LOF variants are of particular interest because they can give rise to distinct phenotypes in the same gene via contrasting molecular mechanisms [2]. For example, GOF mutations in the *STAT1* gene cause Chronic mucocutaneous candidiasis (CMC)—a susceptibility to Candida infection of the skin, nails, and mucous membranes [2]. By contrast, LOF variants in *STAT1* result in Mendelian Susceptibility to Mycobacterial Disease (MSMD)—an immunodeficiency characterized by vulnerability to weakly virulent mycobacteria [2]. Given the established heterogeneity in phenotypic outcomes and their diverse modes of action, it is necessary to distinguish between GOF and LOF variants to develop a greater understanding of the genetic mechanisms of human disease, estimate individual genetic disease risk, identify candidate drug targets, and construct effective treatment regimens.

To date, effective, practical methods for distinguishing GOF and LOF variants are lacking. Experimental techniques are capable of accurately detecting GOF and LOF variants, but these methods are constrained by their significant cost and low throughput [3]. Rapid computational methods for assessing various aspects of variants such as pathogenicity or impact on protein structure/function have been developed [4–8]. For example, CADD [4] leverages a range of functional annotations and conservation metrics to rank the relative deleteriousness of variants. PolyPhen-2 [5] and SIFT [6] combine the physical characteristics of proteins with evolutionary features such as sequence conservation to predict whether a variant will impact protein structure or function. Tools such as REVEL [7] and BayesDel [8] combine the outputs of other predictors to generate a meta-score indicating variant pathogenicity. Yet, none of these tools have been designed for GOF and LOF classification. Some methods for the prediction of GOF and LOF variants have been reported, but they are limited by restriction to a handful of proteins [9] or lack precomputed predictions or available high-throughput implementations [10] barring them from usage genome-wide.

Here we present LoGoFunc—a robust genome-wide predictor of variant functional impact—and generate predictions of functional outcomes for all missense variants in canonical human transcripts. LoGoFunc is a machine learning model comprising an ensemble of LightGBM [11] classifiers trained on pathogenic GOF and LOF variants identified in the literature. We collected 474 descriptors for use in the model including features derived from AlphaFold2 [12] (AF2) predicted protein structures, graph-based learning-derived network features representing interactions within the human protein interactome, measures of evolutionary constraint and conservation, and many others. We analyze the distributions of these features across GOF, LOF, and neutral variants, highlighting structural and functional features of proteins as well as features related to disease mechanisms such as splice disruption. Next, we assess LoGoFunc’s performance and demonstrate that LoGoFunc generates state-of-the-art predictions of GOF, LOF, and neutral variants and is better able to distinguish between pathogenic GOF and LOF variants than tools trained solely to predict pathogenicity or general variant impact. Finally, we investigate which features most influence LoGoFunc’s predictions and identify relationships between high confidence, predicted GOF and LOF variants, and patient phenotypes. We provide precomputed GOF, LOF, and neutral predictions for missense variants genome-wide, which are freely available for rapid retrieval and analysis at https://itanlab.shinyapps.io/goflof/ [13].

## Methods

### Dataset assembly

We obtained 11,370 labeled pathogenic GOF and LOF variants from Bayrak et al. [14]. To supplement this dataset, we collated the 65,075 variants that were deposited in the Human Gene Mutation Database [15] (HGMD) Professional version 2021.3 database specifically in 2020 and 2021 and assigned labels using the strategy employed by Bayrak et al. [14]. From these variants, we first selected 32,911 disease-causing class (DM) variants. We then used the Spacy 3.0.6 NLP library to search for GOF- and LOF-related nomenclature in associated publications for each DM variant. Using the phrase-based matching algorithm PhraseMatcher, we iteratively searched the paper titles and abstracts from all associated publications for the patterns “gain of function(s)”, “gain-of-function(s)”, “GOF”, “loss of function(s)”, “loss-of-function(s)”, and “LOF” with text converted to lowercase to allow for case sensitivity. When at least one of the publications indicated GOF or LOF, we labeled the corresponding variant accordingly. When there was a disagreement, i.e., a variant was found as GOF in one abstract and LOF in another abstract, the variant was excluded from the dataset. NLP-derived labels were checked manually by examining the literature for a subset of variants from the dataset. Based on the results of this analysis, the true positive rate of the NLP labeling approach was estimated to be about 90% [14]. Additionally, we downloaded all variants from ClinVar with the “Pathogenic” and “Likely Pathogenic” significance designations along with the associated PubMed IDs when provided by ClinVar (version 2023–08-13) [16]. Variants without an accompanying citation could not be labeled via our NLP procedure and were thus filtered. Following the labeling procedure described above, we retrieved the abstracts associated with the ClinVar variants and applied our NLP pipeline to extract terminology denoting GOF and LOF. We then associated labels derived from these abstracts with the respective variants. As a result, we were able to procure an additional 14,251 LOF variants and 823 GOF variants not included in our HGMD-derived dataset; 15,562 variants from ClinVar with the “Benign” significance designation and matched to genes from which came the ClinVar GOF and LOF variants were also selected.

Putatively neutral variants were selected from the gnomAD v2.1 [17] exome sequencing data. gnomAD variants were selected from genes represented by the labeled GOF and LOF variants after filtering HGMD pathogenic variants from the gnomAD dataset. gnomAD variants were not filtered by minor allele frequency or other features to avoid selecting a biased sample. A minimum of two gnomAD variants and up to the number of GOF or LOF variants, whichever was the lower, were selected from each gene represented by the labeled GOF and LOF variants for a total of 13,361 putatively neutral variants. The complete labeled dataset comprising 1492 GOF, 13,524 LOF, and 13,361 neutral variants was split into training and testing sets such that the ratio of GOF to LOF to neutral variants in the training and testing sets reflected the ratio in the complete dataset and such that there was no overlap of represented genes between the training and testing sets (Additional file 1: Table S1). The training set and testing sets comprise 90% and 10% of the complete dataset, respectively. All optimization and selection of preprocessing steps, model architecture, and model hyperparameters were performed via nested cross-validation on the training set, while the testing set was used exclusively for the assessment of the model. To investigate the impact of homology on the model, we constructed an alternative dataset split with the additional stipulation that proteins represented by variants in the training set share no more than 40 percent sequence similarity with proteins represented by variants in the testing set [18]. Additionally, the homology-disjoint training and testing sets were filtered such that only one representative protein from each homology cluster remained. When variants from homologous proteins were identified only those from the protein with the greatest number of labeled samples were retained for calculating performance. Sequence identity was determined via the CD-HIT [19] tool using protein amino acid sequences from the Ensembl database version 106.

### Variant annotations

A wide variety of features, putatively correlated with variant functional effects, were obtained for use in the classifier. These features were influenced by previous methods for variant effect classification [4, 14, 20] and were chosen to maximize LoGoFunc’s power for classifying pathogenic GOF and LOF. Features were selected whenever possible from datasets and methods with high genome/proteome coverage, and some redundancy between the collected features was allowed, considering the potential for varying quality and coverage among the different sources. To leverage the recent availability of high-quality protein models from AF2 across the proteome, several tools for the calculation or prediction of protein structural qualities, such as ligand-binding and residue contacts, were employed.

Ensembl’s Variant Effect Predictor [21] (VEP) version 106 was employed to annotate all variants according to their GRCh38 genomic coordinates. VEP provided affected transcripts, genes, and proteins, and the position of variants within these elements where applicable. VEP[21]plugins provided pathogenicity predictions from CADD [4], SIFT [6], PolyPhen2 [5], and CONDEL [22]. Additional pathogenicity predictions were collected using the VEP dbNSFP [23] plugin version 4.1a, along with variant allele frequencies, and conservation scores from PhastCons [24], PhyloP [24], SiPhy [25], and GERP +  + [26]. VEP plugins were also used to retrieve BLOSUM62 [27] scores, GERP [28] scores, distances from variants to the nearest exon junction boundary and the nearest transcription start site, MaxEntScan [29] predictions, dbscSNV [30] splice variants, and predictions of variants allowing for transcript escape from nonsense-mediated decay. AF2 [12] structural models were downloaded from the AlphaFold Protein Structure Database version three [31]. The Biopython PDB module was used to load Protein Data Bank [32] formatted AF2 models and to calculate various geometric properties of proteins and residues. Specifically, residue contacts were inferred when the α-carbons of a given pair of residues resided within 12 Angstroms of each other in 3D space. Similarly, the distance of each residue from the protein center of mass was defined as the 3D distance in Angstroms from the residue’s α-carbon to the protein center of mass as calculated by the Biopython PDB module. To calculate the number of proximal HGMD pathogenic and gnomAD variants in a residues 3D environment, we first mapped protein coordinates to genomic positions for the 18,901 canonical human proteins for which UniProt [33] provides such a mapping. The number of all pathogenic or gnomAD variants, regardless of molecular consequence, occurring in the nine closest residues in 3D space based on the structural models was then summed for each residue in each protein. The Biopython PDB and DSSP [34] modules were used to extract secondary structure characterizations and relative solvent accessibility (RSA) for the model residues. Putative protein–ligand binding sites were predicted using ConCavity [35] v0.1 with the protein structural models as input (default parameters). DDGun [36] and GraphBind [37] were similarly employed to predict variant impacts on protein stability and ligand binding residues respectively using the default parameters and the structural models. Because the pLDDT score, which indicates AF2’s confidence in its prediction of each residue, has been demonstrated to correlate with protein secondary structure, residues with lower pLDDT scores were not excluded when generating features from AF2 models [38]. The protein–protein interaction (PPI) network from the STRING v11 database was processed with node2vec, a random-walk based algorithm for representational learning on graphs/networks [39, 40]. Node2vec reduced the dimensionality of the PPI to 64 vectorial features representing the qualities of each individual protein in the PPI as a function of its interactors. All other features were collected from their respective web servers or calculated via standalone tools (Additional file 2, Additional file 1: Table S2).

### Feature analysis and feature importance

Feature enrichments were calculated via Fisher’s exact test. Continuous features obtained from the DescribePROT [41] database were categorized according to the cutoffs derived from proteome-wide metrics described in Zhao et al. [41]. Residues were classified as buried if their RSA was less than 20%; otherwise, they were regarded as exposed. Grantham [42] scores for amino acid substitutions were considered to be conservative if lower than 100 and radical if greater than or equal to 100. The numbers of residue contacts were binned into categories “high” and “low” based on the median number of residue contacts across the 20,504 proteins included in the AF2 *Homo sapiens* reference proteome dataset. Similarly, the number of residue proximal pathogenic variants from the HGMD and residue proximal gnomAD variants were categorized as “high” or “low” based on the median value of each of these features across the 18,901 proteins for which UniProt provided a mapping between genomic coordinates and residue position. Other continuous features were categorized by assigning a cutoff according to the value recommended by the authors of the tools from which the features were derived. When no such cutoff was reported, a cutoff of 0.5 was selected for probabilistic features. Distance from exon–intron junction boundaries and MMSplice [43] predictions were compared via one-sided two-sample *t*-tests. The Benjamini–Hochberg correction [44] was applied at an alpha level of 0.05 to control for false positives as a result of multiple testing. Feature importance was assessed via the SHAP [45] Python package version 0.41.0. Specifically, the mean SHAP values across the ensembled LightGBM [11] models were generated via the SHAP tree explainer model.

### Preprocessing of input data

Preprocessing steps were applied to prepare sample variants for prediction. An ordinal encoder was fitted to the categorical features in the training set and used to encode the categorical features in the training and test sets. Missing values were imputed either with a constant (− 1) or with the median value of the feature in the training set. Zero variance features in the training set were dropped from both the training and test sets. Finally, random oversampling was performed on the GOF and neutral variants to bring their total count in the training set equal to the majority class, LOF.

### Model selection

We performed fivefold outer, fivefold inner, nested cross-validation in which folds did not contain variants from the same sets of genes on the training dataset to assess the variance associated with our preprocessing pipeline, model hyperparameters, and model architecture (Additional file 2: Fig. S1). Specifically, we evaluated the performance and generalizability of four models: RandomForest [46], LightGBM, XGBoost [47], and Neural Networks. For each algorithm, the data preprocessing procedure and relevant hyperparameters were tuned for 200 rounds in each iteration of the inner cross-validation loop with the Optuna [48] optimization library to maximize the macro-averaged F1-score (F1) (for hyperparameter search spaces see Additional file 2). The F1 score is a function of the precision and recall, defined as follows, where *y* is the set of predicted samples, label pairs, and *y’* is the set of true sample, label pairs:

$$\mathrm{precision}\left(y,y{\prime}\right)=\frac{\left|y\cap y{\prime}\right|}{\left|y\right|}$$

$$\mathrm{recall}\left(y,y{\prime}\right)=\frac{\left|y\cap y{\prime}\right|}{\left|y{\prime}\right|}$$

$$F1\left(y,y{\prime}\right)=\frac{2\times \left(\mathrm{precision}\left(y,y{\prime}\right)\times \mathrm{recall}\left(y,y{\prime}\right)\right)}{\left(\mathrm{precision}\left(y,y{\prime}\right)+\mathrm{recall}\left(y,y{\prime}\right)\right)}$$

To extend the F1 score to multiclass classification, we calculated the macro-averaged F1-score, defined as follows where *L* is the set of labels:

$$F{1}_{\mathrm{macro}}=\frac{1}{\left|L\right|}{\sum }_{l\in L}y{^{\prime}}_{l}F1\left({y}_{l},y{^{\prime}}_{l}\right)$$

The preprocessing pipeline and hyperparameters that performed best for each model in the inner cross-validation iteration were then used to assess each model on the held-out set of the outer cross-validation loop. After all rounds of outer and inner cross-validation, the median Matthew’s Correlation Coefficient (MCC) and F1 were compared to determine which model performed best for the dataset. The MCC is defined as follows where *k* is the number of classes and *kl* refers to an element of the confusion matrix:

$$MCC=\frac{\sum_k \sum_l \sum_m {C}_{kk}{C}_{lm}-{C}_{kl}{C}_{mk}}{\sqrt{\sum_k \left(\sum_l {C}_{kl}\right)\left(\sum_{k'|k' \neq k} \sum_{l'} {C}_{k{\prime}l{\prime}}\right)}\sqrt{\sum_k \left(\sum_l {C}_{lk}\right)\left(\sum_{k'|k' \neq k} \sum_{l'} {C}_{l{\prime}k{\prime}}\right)}}$$

LightGBM obtained the best MCC and F1 scores across outer folds (Additional file 2: Fig. S2). We subsequently performed the same nested cross-validation procedure described above with ensembles of 5 to 31 LightGBM models with individual model hyperparameters and the number of ensemble estimators tuned simultaneously. The ensembled LightGBM models achieved the highest MCC and F1 scores across outer folds and were selected as the final model. Subsequently, we performed the inner cross-validation procedure with all of the training data to determine the final number of ensemble estimators and model hyperparameters.

The final LightGBM classifiers, which are gradient-boosted decision trees, are subjected to significant L_1_ and L_2_ regularization as well as constraints on maximum tree depth and impurity reduction as per the hyperparameters selected during cross-validation. Further, decision tree algorithms are generally robust to overfitting as a result of multicollinearity among features [49]. Together, these qualities combat potential overfitting due to the relatively large feature set employed by LoGoFunc.

### LoGoFunc performance

LoGoFunc’s performance was assessed via average precision (AP), F1, and MCC calculated using scikit-learn version 1.1.1. AP is defined as follows, where *n* is the *n*_th_ threshold:

$$AP={\sum }_{n}\left({\mathrm{recall}}_{n}-{\mathrm{recall}}_{n-1}\right){\mathrm{precision}}_{n}$$

For each class, we computed these metrics as one vs. rest tasks where the class in question was relabeled as one and the other classes were relabeled as zero.

### Gene 95% confidence intervals

For each variant class, GOF, LOF, and neutral, we selected predictions from the training and testing sets for variants of that class. We applied the Kolmogorov–Smirnov [50] test for goodness of fit to predictions for these variants with continuous distributions implemented in scipy [51] version 1.0.1. For each distribution, we first estimated the distribution parameters that best modeled the predictions using scipy and then selected the parametrized distribution with the highest *p*-value from the Kolmogorov–Smirnov test; 95% confidence intervals were then calculated using the best fitting, parameterized distribution for predictions from each class respectively and clipped between zero and one where applicable. When five or more variants were available from a given class for a given gene, we repeated the above process to calculate gene-specific 95% confidence intervals. When fewer than five variants were available for a class in a gene, we defaulted to the 95% confidence intervals calculated for predictions from the entire dataset.

### Method comparison

LoGoFunc was compared to other computational methods by assessing the AP. All GOF (n. 136) and LOF (n. 545) variants from the test set for which all compared tools provided a prediction of pathogenicity were collected. APs were calculated, treating GOF as the positive class. To assess the performance separating neutral variants from GOF and LOF, we added all neutral (n. 411) variants from the test set for which each tool provided a prediction. APs were again calculated, this time with GOF and LOF variants as the positive classes respectively, and neutral as the negative class. Finally, we calculated the one-vs.-all APs with GOF and LOF variants as the positive class and neutral variants as the negative class. Most of the compared tools provide predictions in which higher scores correspond to a greater likelihood that a given variant will be damaging. However, SIFT outputs predictions between zero and one in which lower scores correspond to a greater likelihood of a damaging effect. LoGoFunc’s neutral prediction is a value between zero and one, where higher scores indicate a greater likelihood of neutrality. Thus, to ensure consistency between all compared tools when treating neutral as the negative class and GOF and LOF as the positive class, SIFT and LoGoFunc neutral predictions were transformed by subtracting each prediction from one before assessing AP.

### PheWAS of predicted GOF and LOF variants

The Mount Sinai BioMe BioBank comprises de-identified whole exome sequencing (WES) data from two distinct cohorts. The first WES cohort includes 30,813 samples, which were sequenced on the Illumina v4 HiSeq 2500 system following the extraction of exome regions using the IDT xGen capture platform. The second WES cohort includes 14,985 BioMe participants who were sequenced on the Illumina NovaSeq6000 sequencing system with the Agilent SureSelect QXT Human All Exon V7 targeting kit.

For our initial Phenome-Wide Association Studies (PheWAS), we utilized the data from the first WES cohort, specifically including the information of 29,477 participants for whom we had access to both WES data, demographic information, and associated ICD-10 electronic health records (EHR). We mapped ICD-10 codes to 1856 phecodes using Phecode Map 1.2 [52]. Subsequently, we selected 1075 phecodes with at least 50 cases for further analysis.

A phenome-wide association study (PheWAS) was conducted employing the PLINK 2.0 [53] glm function with the Firth regression model, a robust method for association testing of rare variants in cohorts characterized by skewed case–control ratios [54, 55]. Our analysis included age, biological sex, and the first ten principal components as covariates. Principal component analysis (PCA) was performed using PLINK 2.0, with the exclusion of variants deviating from Hardy–Weinberg equilibrium (*P* < 1 × 10^−6^) on linkage disequilibrium (LD)-pruned autosomal variants (*r*^2^ > 0.2, window size 50, and step size 5) with a minor allele frequency greater than 0.05. We excluded association results with a minor allele count (MAC) < 20 and those with a convergence error. To account for multiple testing, we used a Bonferroni-corrected *P* threshold of 4.65 × 10^–5^ (0.05/1075). Additionally, we assessed the independence of predicted and previously identified GOF and LOF variants using PLINK 1.9 with the –r2 option.

For the replication of significant associations identified in the initial PheWAS for the predicted GOF and LOF variants, we leveraged data from the second WES cohort, comprising 14,985 participants for whom we had access to both WES data, demographic information, and associated ICD-10 EHR. The same methodology applied in the initial PheWAS was used for the replication, including the mapping of ICD-10 codes to phecodes, PCA, and association testing. However, due to the smaller sample size in the replication cohort, we adjusted the test criteria by setting the minimum number of cases to 20 and the minimum MAC to 10 to ensure statistical robustness.

## Results

### Labeled GOF, LOF and neutral variant dataset curation

**Fig. 1:**
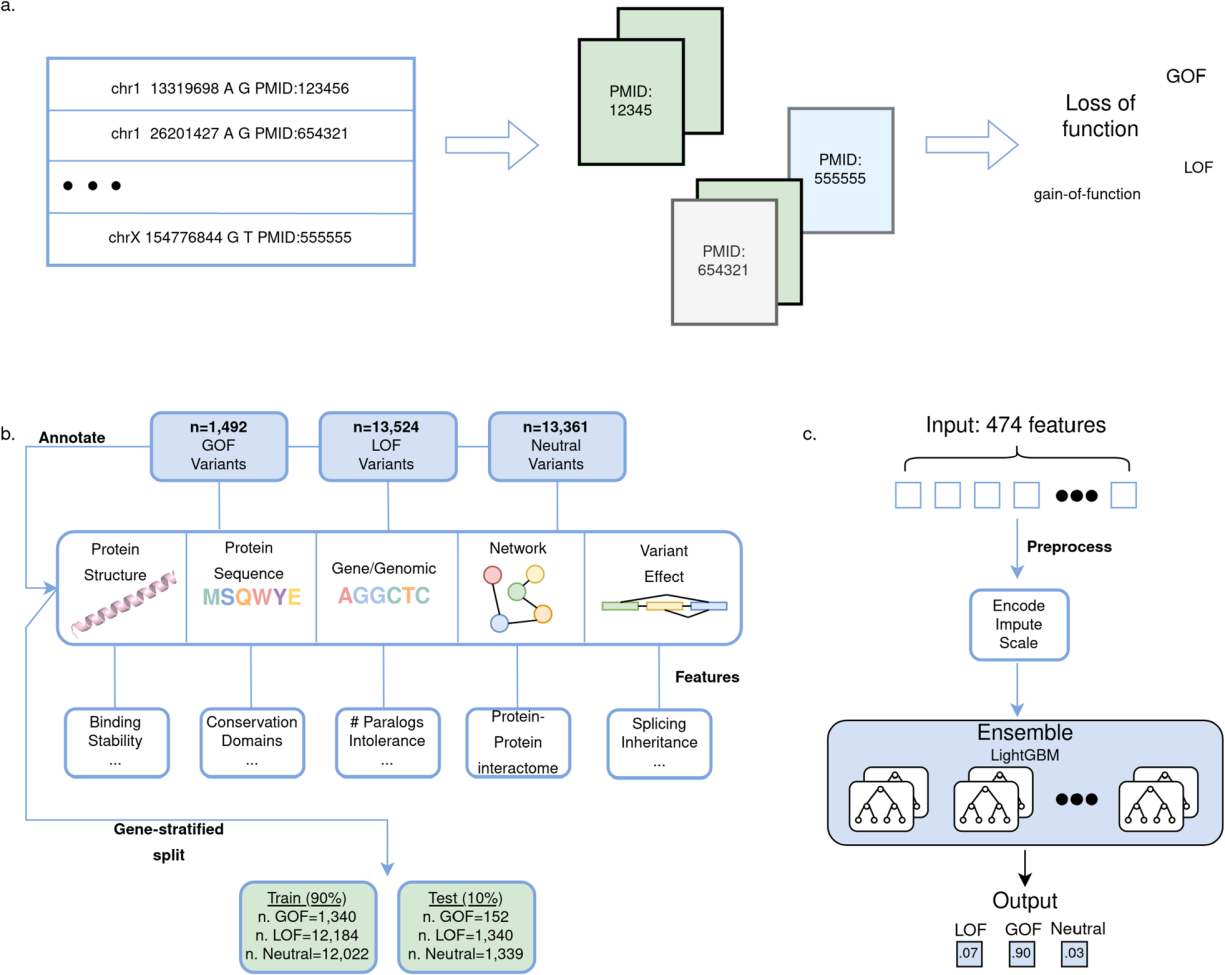
LoGoFunc workflow and model architecture. **a** Pipeline for the collection of labeled pathogenic GOF and LOF variants. Related abstracts for high confidence pathogenic variants from the HGMD [15] were searched for nomenclature denoting gain or loss of function. **b** Dataset preparation and annotation. 1492 GOF, 13,524 LOF, and 13,361 neutral variants were obtained from the GOF/LOF database [14], HGMD, and gnomAD [17]. Using VEP [21] and other tools, variants were annotated with protein structural and functional features derived from AlphaFold2 [12] models or from sequence, with gene- and genomic-level features, variant-level features, and network-derived protein interaction features. The annotated data were split into training and test sets comprising 90% and 10% of the dataset respectively, stratified by variant label. **c** Model architecture and output. Variants are input to the model represented as an array of the 474 collected features. These features are encoded, imputed, and scaled prior to prediction. The model consists of an ensemble of 27 LightGBM [11] classifiers. A probability is output for each class, GOF, LOF, and neutral

LoGoFunc was trained on a dataset of pathogenic GOF and LOF variants, collected from the literature via a NLP pipeline [14]. In brief, the NLP pipeline parses abstracts associated with high-confidence, disease-causing variants derived from the HGMD [15]. Professional version 2021.3, searching for terminology denoting GOF and LOF (Fig. 1a). In total, 1492 GOF variants from 344 genes and 13,524 LOF variants from 2030 genes were collected and labeled. In addition, 13,361 putatively neutral variants were randomly selected from the genes in which the labeled GOF and LOF variants occur, from gnomAD v2.1 [17] exome sequences (Fig. 1b, Additional file 1: Table S1). We used Ensembl’s VEP [21] to map the genomic coordinates of each variant to impacted genes and proteins where applicable and to retrieve molecular positioning information (e.g., residue position, transcript position) for each variant in the dataset. Leveraging this positional information, we further annotated each variant with 474 different features (Additional file 1: Table S2). These include protein structural features such as residue solvent accessibility and total residue contacts calculated from AF2 [12] predicted protein structures, gene-level features such as gene haploinsufficiency, variant-level features including splicing effects and inheritance patterns, and network features encapsulating the STRING [39] protein–protein interaction (PPI) network, representing, as a low-dimensional vector of numerical values, the qualities of a given protein’s inter-protein interactions in the context of its interacting partners (Fig. 1b). The annotated variants were split into label-stratified, gene-disjoint training and testing sets comprising 90% and 10% of the full dataset, respectively (Fig. 1b, Additional file 1: Table S1).

### GOF, LOF and neutral variants stratified by protein features

**Fig. 2:**
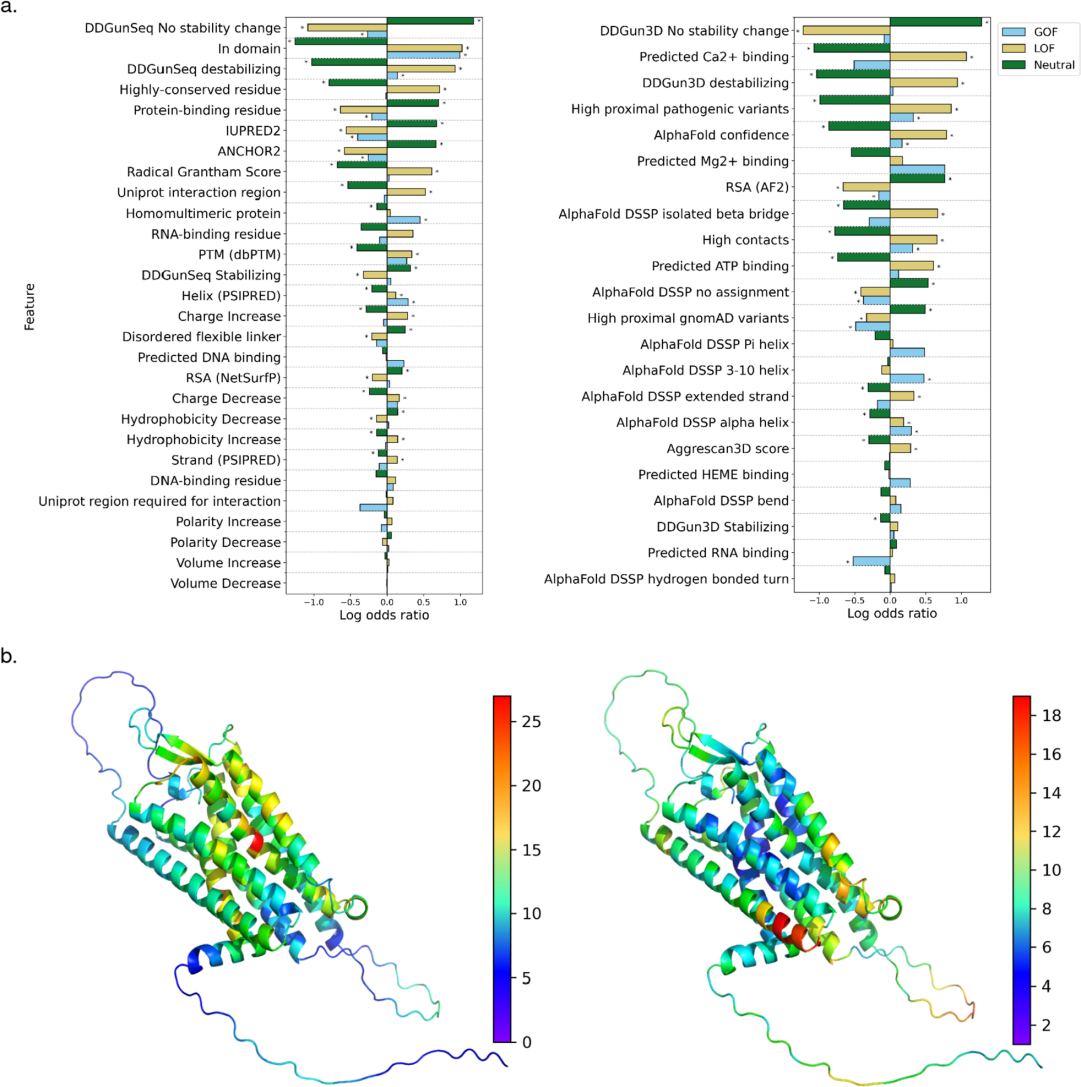
Structure- and sequence-based protein feature analysis. **a** Enrichments and depletions for protein structural and functional features used by the LoGoFunc model. GOF (blue), LOF (orange), and neutral (green) log odds ratios are displayed for each feature. Significant enrichments and depletions are denoted by asterisks. Significance was calculated with Fisher’s exact test, Benjamini–Hochberg corrected [44] to allow for multiple comparisons. (Left) Features derived from protein sequences or protein interaction data. (Right) Features derived from AlphaFold2 [12] protein structures. **b** AlphaFold2 predicted structure of the Vasopressin V2 receptor protein. (Left) Residues colored by the number of HGMD [15] pathogenic variants occurring in the nine closest neighboring residues in space. (Right) Residues colored by the number of gnomAD [17] variants occurring in the nine closest neighboring residues in space

We postulated that structural and functional features of proteins predicted or derived from protein sequences and AF2 structural models may help to stratify GOF, LOF, and neutral variants. To investigate the varying impact on protein structure and function as well as potential differential localization within distinct protein regions, we examined protein features by calculating enrichments for each variant class, determined via Fisher’s exact test (Fig. 2a, Additional file 1: Table S3, 4). In total, GOF, LOF, and/or neutral variants demonstrated significant enrichments or depletions across 17 features derived from AF2-predicted protein structures and across 20 protein features derived from protein sequences or otherwise describing the proteins (Fig. 2a, Additional file 1: Table S3, 4). For example, LOF variants were significantly more likely to have a destabilizing effect on proteins, as predicted by DDGun [36], and to occur in highly conserved residues as determined by multiple sequence alignments generated by MMSeqs2 [56] (Fig. 2a, Additional file 1: Table S3, 4). GOF variants were found to be significantly more likely to occur in homomultimeric proteins and α-helices among other features (Fig. 2a, Additional file 1: Table S3, 4). Interestingly, both GOF and LOF variants were significantly more likely to have a high number of pathogenic HGMD variants in their spatial proximity (“Methods”), whereas neutral variants were significantly more likely to have a high number of gnomAD variants in their immediate vicinity. This phenomenon is exemplified by the Vasopressin V2 receptor protein in which pathogenic and putatively neutral variants can be qualitatively observed to localize to distinct regions of the 3D AF2 protein structure (Fig. 2b). Finally, neutral variants were significantly enriched for several features including occurrence in disordered protein regions and significant depletion in Pfam [57] or InterPro [58] domains among other features (Fig. 2a, Additional file 1: Table S3, 4).

We additionally performed Fisher’s exact test with neutral variants excluded so as to compare only pathogenic GOF and LOF variants and noted significant differences between GOF and LOF variants for seven structure-associated features and seven sequence or otherwise associated features (Additional file 2: Fig. S3, Additional file 1: Table S5, 6). Interestingly, GOF variants were enriched and LOF variants were depleted in Pfam or InterPro domains, in α-helices, in homomultimer-forming proteins, and for residues not affecting protein stability based on sequence-based and structural evidence (Additional file 2: Fig. S3, Additional file 1: Table S5, 6). Conversely, we found that LOF variants were enriched for destabilizing amino acid substitutions, for highly conserved residues and radical Grantham [42] position-specific scoring matrix substitutions, for high AF2-predicted local distance difference test scoring (pLDDT) residues, and in β-strands (Additional file 2: Fig. S3, Additional file 1: Table S5, 6).

### Training, architecture and performance of LoGoFunc

To predict pathogenic GOF, pathogenic LOF, and neutral variants, we developed LoGoFunc, a soft-voting ensemble composed of 27 LightGBM [11] classifiers. Variants are represented as an array of 474 features that are encoded, imputed, and scaled before being input to the model which outputs three values corresponding to the predicted probability that the input variant results in a GOF, LOF, or neutral phenotype, respectively (Fig. 1c).

**Fig. 3:**
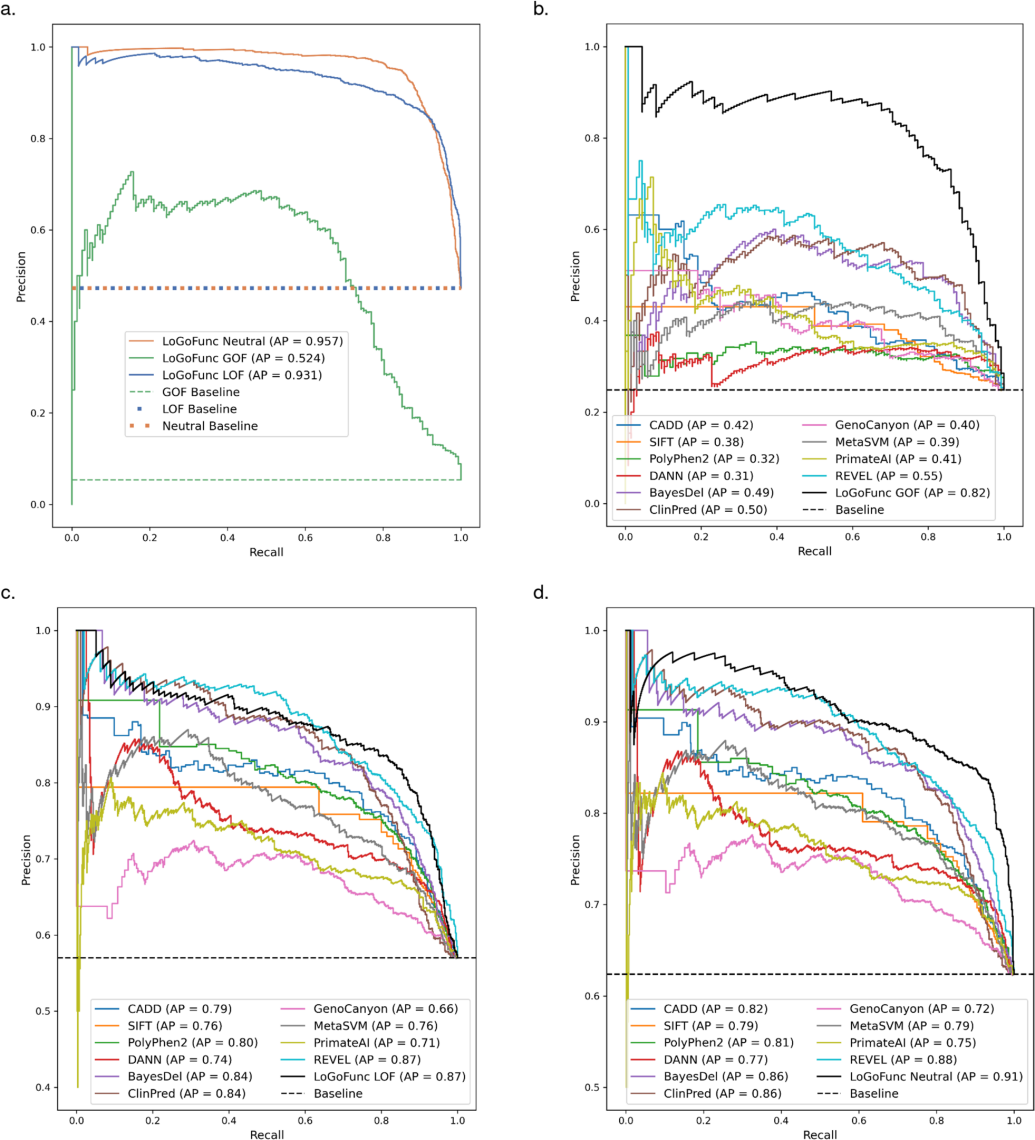
Performance assessment. Precision-recall curves indicating the discriminatory power of various pathogenicity prediction methods and LoGoFunc on a set of variants from the test set for which predictions were available from all compared tools. **a** LoGoFunc’s performance on all testing variants (n. GOF = 152, n. LOF = 1340, n. neutral = 1339). **b** GOF (n. 136) vs. neutral (n. 411). **c** LOF (n. 545) vs. neutral (n. 411). **d** GOF (n. 136) and LOF (n. 545) combined vs. neutral (n. 411)

LoGoFunc achieved notable success in classifying GOF, LOF, and neutral variants. Considering the class imbalance in the dataset, we calculated the AP scores on the held-out testing data for each class. As expected, predicting GOF variants proved to be the most challenging task as GOF variants were the least represented in the training dataset. However, LoGoFunc still performed well with AP values of 0.52, 0.93, and 0.96 for GOF, LOF, and neutral variants, respectively (Fig. 3a). We also calculated the F1-score and MCC for LoGoFunc’s predictions of variants from each class. LoGoFunc realized F1-scores of 0.56, 0.87, and 0.89 and MCCs of 0.54, 0.75, and 0.80 for GOF, LOF, and neutral variants, respectively. To assess the impact of variants from homologous proteins in the training and testing sets on the model’s performance, we retrained the model on a training dataset that was constructed such that protein sequence identity with proteins in the testing set was no more than 40 percent. When testing on this sequence unique testing dataset the model achieved AP values of 0.37, 0.92, and 0.96 (Additional file 2: Fig. S4), F1 scores of 0.37, 0.84, and 0.88, and MCCs of 0.34, 0.70, and 0.79, for GOF, LOF, and neutral variants, respectively. To aid in the interpretation of LoGoFunc’s predictions, we calculated 95% confidence intervals for determining cutoffs for each class, as well as 95% confidence intervals for determining GOF, LOF, and neutral prediction cutoffs per gene (Additional file 1: Table S7).

We further assessed LoGoFunc on an independent set of variants collected from ClinVar for which we were able to derive functional labels from the literature. In total, we collected 823 GOF, 14,251 LOF variants, and 15,562 neutral variants from ClinVar which did not occur in our dataset collected from the HGMD and gnomAD. We found LoGoFunc to perform well on the ClinVar variants, achieving AP values of 0.52, 0.98, and 0.99, respectively, for GOF, LOF, and neutral variants (Additional file 2: Fig. S5a). Similarly, LoGoFunc realized F1 scores of 0.51, 0.90, and 0.93 and MCCs of 0.52, 0.94, and 0.96 for GOF, LOF, and neutral variants, respectively.

### Benchmark against variant assessment algorithms

Current methods for classifying pathogenic GOF and LOF variants are limited by a restriction to a small number of proteins or have low predictive accuracy [14]. We therefore compared LoGoFunc to ten established predictors of pathogenicity/deleteriousness: CADD [4], SIFT [6], PolyPhen2 [5], DANN [59], BayesDel [8], ClinPred [60], GenoCanyon [61], MetaSVM [62], PrimateAI [63], and REVEL [7]. Importantly, none of these methods were developed to discriminate between pathogenic GOF and LOF, but rather were developed or are used to estimate the pathogenicity of genetic variants in general. To equitably assess each method’s ability to classify the different classes of pathogenic variants in our dataset, we selected the subset of 1092 GOF, LOF, and neutral variants from the test set for which all predictors provided a score. Of these variants, 136 were GOF, 545 were LOF, and 411 were neutral. Importantly, these variants are all missense, as the majority of compared methods provide predictions only for missense variants. We tested each method’s performance in classifying the pathogenic GOF and neutral variants, the pathogenic LOF and neutral variants, and all pathogenic and neutral variants separately. Finally, we examine if these methods produce scores that can discriminate pathogenic GOF from pathogenic LOF variants. Unsurprisingly, when comparing the methods for separating GOF and LOF variants, LoGoFunc is the only method to achieve a substantial improvement over the baseline with an AP of 0.63 followed by GenoCanyon with a score of 0.25 (Additional file 2: Fig. S6). For separating pathogenic GOF and neutral variants, LoGoFunc achieved an AP of 0.82 (Fig. 3b) and an AP of 0.87 for pathogenic LOF vs. neutral variants (Fig. 3c). The next best tool, REVEL, achieved AP values of 0.55 and 0.87 for GOF and LOF vs. neutral, respectively (Fig. 3b,c). Finally, we calculated the one-vs.-all AP for the neutral variants against the GOF and LOF variants. Once again, LoGoFunc scored highest with an AP of 0.91, followed by REVEL with an AP of 0.88 (Fig. 3d). We additionally compare the performance of these methods on the sequence unique testing set (Additional file 2: Fig. S7), though it should be noted that this comparison may overestimate the performance of the compared methods in relation to LoGoFunc as they may have been trained on variants in our testing set and were likely trained on an overlapping and/or homologous set of genes.

Previously, two methods, funNCion [9] and VPatho [10], were developed for the classification of GOF and LOF variants. However, both are limited in their applicability; in the case of funNCion, by a restriction to a small set of ion channel proteins, and in the case of VPatho, by an inability to produce discriminative predictions. We compared LoGoFunc to funNCion on the set of 27 GOF and 58 LOF variants in funNCion’s testing set and found our method to compare favorably despite focusing on a broader predictive task. Particularly, when treating GOF as the positive class, LoGoFunc’s GOF score achieved an AP of 0.77 compared to funNCion’s AP of 0.58 (Additional file 2: Fig. S8a). When treating LOF as the positive class, LoGoFunc’s LOF score achieved an AP of 0.87 compared to funNCion’s AP of 0.91 (Additional file 2: Fig. S8b). Similarly, we compared LoGoFunc to VPatho on all variants from our testing dataset for which we were able to retrieve predictions from the VPatho website (n. GOF = 152, n. LOF = 1339, n. Neutral = 1339). As above, we compared LoGoFunc and VPatho’s performances for classifying GOF vs. LOF variants (Additional file 2: Fig. S9a), GOF vs. neutral variants (Additional file 2: Fig. S9b), LOF vs. neutral variants (Additional file 2: Fig. S9c), and all pathogenic vs. neutral variants (Additional file 2: Fig. S9d), separately. We found that for each of these tasks, LoGoFunc substantially outperforms VPatho, predictions from which do not improve meaningfully over the estimated random baseline performance.

**Fig. 4:**
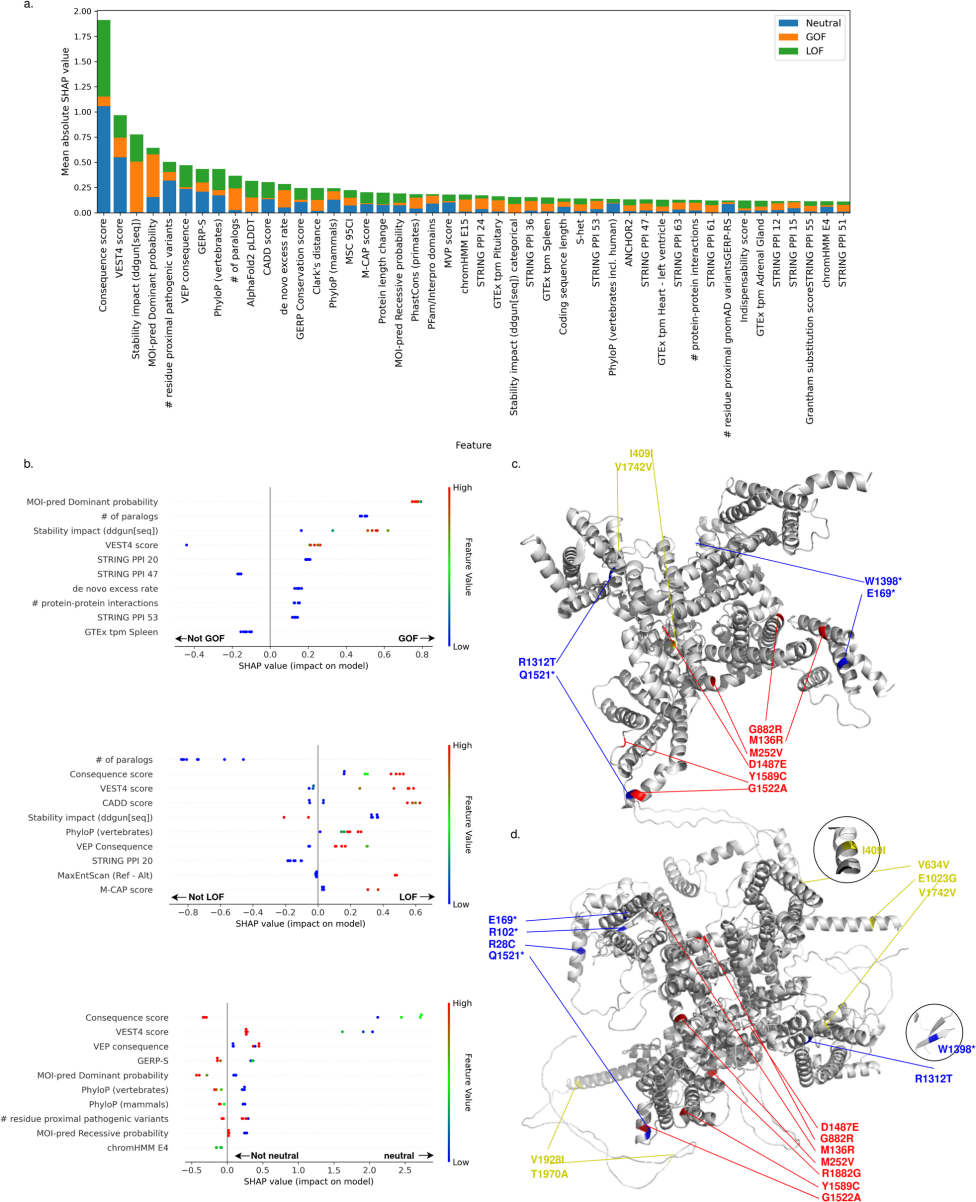
Explanation of LoGoFunc predictions. **a** SHAP values by class for features with combined SHAP values in the 90th percentile and above. **b** (Top) The SHAP values for the top ten features for the seven GOF variants found in the ion channel SCN2A in the test set. (Middle) The SHAP values for the top ten features for the eight LOF SCN2A variants in the test set. (Bottom) The SHAP values for the top ten features for the seven neutral SCN2A variants in the test set. **c** The experimentally determined structure of SCN2A [64] with the represented GOF (red), LOF (blue), and neutral (yellow) SCN2A variants from the test set. **d** The SCN2A model from the AlphaFold2 prediction database annotated with the represented GOF (red), LOF (blue), and neutral (yellow) SCN2A variants from the test set

Because autosomal recessive (AR) disorders are most commonly associated with LOF mechanisms, and GOF variants more commonly associate with autosomal dominant (AD) disorders, we additionally investigated if mode of inheritance predictions—an important feature for the model (Fig. 4a)—alone are predictive of GOF and LOF variants. We selected the 134 GOF, 518 LOF, and 409 neutral variants from our test for which MOI-pred, a mode of inheritance predictor, produced a prediction. Similar to the methods designed for binary classification of variant pathogenicity, we found MOI-pred to be substantially less capable of discriminating between GOF and LOF variants than LoGoFunc (Additional file 2: Fig. S10) and likewise less able to discriminate between GOF and neutral and LOF and neutral variants, respectively.

### LoGoFunc leverages diverse biological signals for prediction

To gain further insight into the model’s performance, we estimate the impact of each included feature on LoGoFunc’s predictions with SHAP [45]—a game theoretic approach for the derivation of explanations for machine learning models (Fig. 4a). We observed that LoGoFunc learned from a diverse array of features describing the genes and proteins containing variants and the variant impact upon these elements. These included functional, conservation, structural, and systems-based/network features, among others (Additional file 1: Table S8, Fig. 4a). For example, the top feature across classes was the consequence score collected from the CADD database of variant annotations which describes the severity of a variant according to sequence ontology [65] consequence terms (Fig. 4a). Other important variant features include predictions indicating pathogenicity from CADD, VEST4 [66], M-CAP [67], and MVP [68], the MOI-pred [69] mode of inheritance prediction of variants underlying autosomal dominant (AD) and autosomal recessive (AR) disease, and various measures of conservation from tools such as GERP [28], PhyloP [24], and PhastCons [24] (Fig. 4a). Several gene-level features were important for the model including the number of gene paralogs, the de novo excess rate [70], the mutation significance cutoff [71] 95% confidence interval, and the indispensability score [72]—all of which have previously been implicated in the stratification of pathogenic GOF and LOF variants and neutral variants [14] (Fig. 4a). In addition, LoGoFunc’s predictions were influenced by features indicating variant effects on protein structure and function such as the predicted variant impact on protein stability, the number of HGMD pathogenic or gnomAD variants proximal to variant impacted residues in 3D space, AF2 pLDDT scores which indicate AF2’s per-residue prediction confidence, and overlapping Pfam or InterPro domains (Fig. 4a). Notably, PPI network features also had a significant impact on the model. We processed the STRING PPI network using node2vec [40] resulting in 64 tabular features summarizing the human protein interactome weighted by the probability of interaction between each pair of putatively interacting proteins. Several dimensions of the transformed PPI network appeared in the list of top features as determined by SHAP [45] (Fig. 4a).

To further investigate the model’s predictions within genes, we examined the 22 variants included in our test set from sodium voltage-gated channel alpha subunit 2 (SCN2A)—an important transmembrane protein implicated in seizure disorders [73] and autism spectrum disorders [74]. Of these 22 variants, VEP indicated 12 to be missense, 4 to be stop-gains, 2 to be splice donor site variants, 3 to be synonymous, and 1 to be intronic. Twelve of the coding variant positions are included in the experimentally determined structure (PDB identifier 6J8E [64]) (Fig. 4c). Because the other ten variants are located in regions not covered by the structure, we analyzed the structural model generated by AF2 (Fig. 4d), which includes the full-length protein. Remarkably, LoGoFunc successfully classified all seven SCN2A pathogenic GOF variants, all seven SCN2A neutral variants, and six of eight pathogenic LOF variants, misclassifying two LOF variants as GOF. We then examined the top ten features indicated by SHAP to contribute to the model’s predictions for the GOF, LOF, and neutral variants separately (Fig. 4b). Again, we found a mixture of gene, protein, variant, and network features influenced the model’s predictions. Specifically, a range of mode of inheritance predictions (MOI-pred) of variants pathogenic for AD inheritance, scores indicating less protein destabilization (DDGun), and high VEST4 scores among others influenced the model to predict the SCN2A GOF variants as GOF. Similarly, several features prompted the model to predict the LOF variants to be LOF, including scores indicating higher impact on transcripts and downstream products, high VEST4 and CADD scores, scores indicating a greater destabilizing effect on proteins (DDgun), and high vertebrate conservation scores (PhyloP). Notably, high values indicating likely loss of native splice sites [29] contributed to the model’s LOF predictions for two LOF variants, consistent with VEP’s characterization of two of the LOF variants as splice donor site variants. The model’s predictions were most influenced towards neutrality by lower consequence scores, lower VEST4 scores, lower estimates of evolutionary constraint (GERP-S [26]), and vertebrate and mammalian conservation scores (PhyloP), and lower MOI-pred scores among other features.

### PheWAS of predicted GOF and LOF variants

**Fig. 5:**
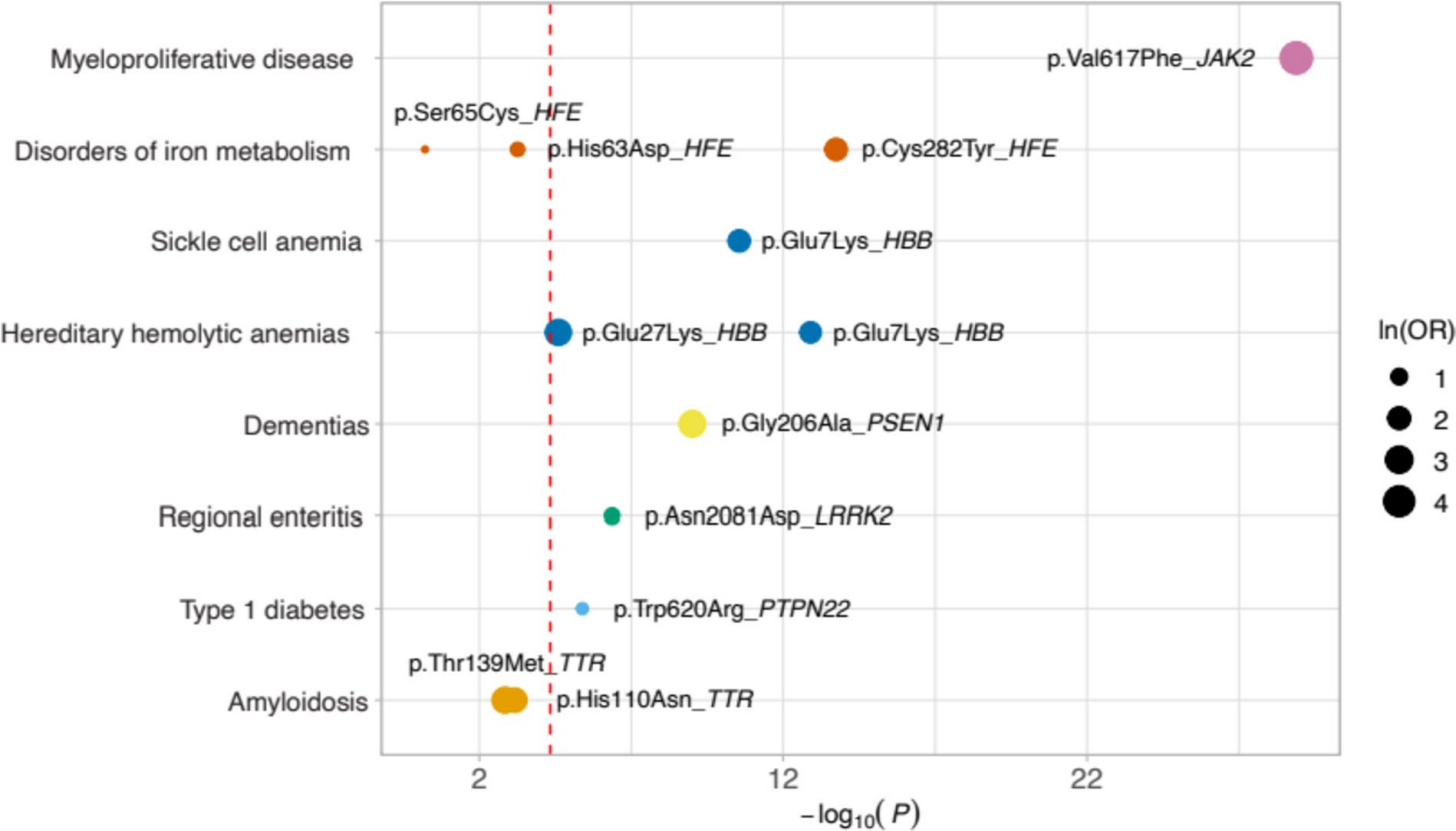
PheWAS of predicted GOF and LOF variants. Selected examples of phenotype associations of predicted GOF and LOF variants in the BioMe BioBank. Each circle represents an association, with the circle size proportional to the natural logarithm of the odds ratios (ln(OR)). The dashed line indicates the Bonferroni-adjusted *P*-value threshold

We conducted phenome-wide association studies (PheWAS) using predicted GOF and LOF missense variants in the BioMe BioBank cohort, comprising 29,477 individuals, which revealed several significant associations (Fig. 5). We compared some of the identified associations with those previously documented for known effects and predicted neutral variants, wherever possible (Additional file 1: Table S9). In summary, we observed a predicted LOF variant in the *HBB* gene, p.Glu7Lys (rs33930165), demonstrating associations with hereditary hemolytic anemias and sickle cell anemia (phecodes = 282 and 282.5, odds ratios [OR] = 5.63 and 6.59, *P* = 1.26 × 10^−13^ and 2.8 × 10^−11^). Similarly, another predicted LOF variant in *HBB*, p.Glu27Lys (rs33950507), showed an association with hereditary hemolytic anemias (OR = 14.8, *P* = 2.51 × 10^−5^). Notably, a previously identified LOF variant in the same gene, p.Glu7Val (rs334), exhibited significant associations with the same phenotypes, with an OR of 16.4 and a *P* value of 9.15 × 10^−103^ for hereditary hemolytic anemias and an OR of 116 with a *P* value of 2.04 × 10^–68^ for sickle cell disease. Importantly, LD analysis indicated that these three variants were independent of each other. In contrast, a predicted neutral variant, p.Ala111Pro (rs10768683), was found to be associated with a reduced risk of sickle cell anemia (OR = 0.38, *P* = 1.2 × 10^–4^).

Another significant association was observed between a known LOF variant in *TTR*, p.Val142Ile (rs76992529), and amyloidosis (phecode = 270.33, OR = 29.9, *P* = 9.07 × 10^–15^). Furthermore, two independent predicted LOF variants in this gene, p.His110Asn (rs121918074) and p.Thr139Met (rs28933981), showed associations with amyloidosis (OR = 10.3 and 14.5, *P* = 6.8 × 10^–4^ and 1.4 × 10^–3^). Conversely, a neutral variant in *TTR*, p.Gly26Ser (rs1800458), did not exhibit an association with amyloidosis (*P* = 0.71). We also identified three predicted LOF variants in *HFE*, namely, p.Cys282Tyr (rs1800562), p.His63Asp (rs1799945), and p.Ser65Cys (rs1800730). While p.Cys282Tyr and p.His63Asp were associated with disorders of iron metabolism (phecode = 275.1, OR = 6.79 and 2.12, *P* = 1.82 × 10^–14^ and 5.5 × 10^–4^), we did not observe such an association for p.Ser65Cys (*P* = 0.61). On the other hand, two predicted neutral variants in *HFE*, p.Arg6Ser (rs149342416) and p.Val295Ala (rs143175221), were not associated with disorders of iron metabolism (*P* = 0.19 and 0.17). Additionally, a predicted LOF variant in *PSEN1*, p.Gly206Ala (rs63750082), exhibited an association with dementias (phecode = 290.1, OR = 17.3, *P* = 9.79 × 10^–10^), while a neutral variant in *PSEN1*, p.Glu318Gly (rs17125721), did not demonstrate such an association (*P* = 0.86).

Moreover, our PheWAS analysis revealed additional interesting associations of predicted GOF variants with various other phenotypes. Notable findings included p.Val617Phe in *JAK2* (predicted GOF, rs77375493), which was associated with myeloproliferative disease (phecode = 200, OR = 40.6, *P* = 1.29 × 10^–29^), p.Trp620Arg in *PTPN22* (predicted GOF, rs2476601), displaying an association with type 1 diabetes (phecode = 250.1, OR = 1.78, *P* = 4.08 × 10^–6^), as well as a predicted GOF variant in *LRRK2*, p.Asn2081Asp (rs33995883), which demonstrated an association with regional enteritis (phecode = 555.1, OR = 2.33, *P* = 4.28 × 10^–7^). Importantly, we successfully replicated the above-mentioned associations of p.Glu7Lys in *HBB*, p.Cys282Tyr in *HFE*, p.Trp620Arg in *PTPN22*, p.Asn2081Asp in *LRRK2,* and p.Val617Phe in *JAK2* in an independent BioMe WES cohort, consisting of 14,985 individuals (Additional file 1: Table S10). We could not replicate the remaining associations due to the limited size of cases or low MAC.

## Discussion

Describing the functional consequences of genetic variations is critical for the development of a better understanding of disease mechanisms. In our previous work [14], we described the curation of a database of pathogenic GOF and LOF variants compiled using a NLP pipeline to extract labels denoting the mechanism of pathogenicity for 11,370 variants from the HGMD. We further annotated these GOF and LOF variants with a variety of features and compared them to identify the distinct biological qualities of variants resulting in these opposing modes of pathogenicity. In the present work, we significantly expand our original database of pathogenic GOF and LOF variants to include an additional 3228 LOF variants and 255 GOF variants from newer releases of the HGMD database. We also expand the set of features describing these variants to include additional measures of conservation, protein structural characteristics including features derived from AlphaFold2 structures, features describing the protein–protein interactome, and others, and we used this set of expanded labeled variants and biological features to develop LoGoFunc, a rapid and accurate classifier of GOF, LOF, and neutral variants. Four key findings emerge from this work.

First, we observe that pathogenic GOF, LOF, and neutral variants inhabit varying structural and functional regions of proteins, exert differing effects on protein structure, and inhabit proteins with different PPI characteristics (Fig. 2, Additional file 2: Fig. S3). Specifically, LOF variants consistently demonstrate a greater propensity for the disruption of protein structure and/or function, as has been previously demonstrated [9, 75, 76]. Particularly, LOF variants are significantly more likely to have a predicted destabilizing effect on protein structure and significantly less likely to stabilize or result in a negligible effect on protein structure (Fig. 2a). LOF variants are enriched for highly conserved residues and for more radical amino acid substitutions (Fig. 2a). Similarly, LOF variants are enriched for known post-translationally modified residues (PTMs) and are more likely to be buried in protein structures. GOF variants compared to LOF, while enriched in potentially functionally important Pfam [57] domains, appear to impact protein structure less radically (Additional file 2: Fig. S3). Indeed, compared to LOF variants, GOF variants were depleted for predicted protein destabilizing substitutions, highly conserved residues, and radical amino acid substitutions (Additional file 2: Fig. S3). Interestingly, when considering both sequence-based predictions and evidence derived from AF2 [12] structures, we found GOF variants to be enriched in α-helices and LOF variants to be enriched in β-strands (Additional file 2: Fig. S3). Previous studies have demonstrated mutations in α-helices to be less structurally impactful than mutations occurring in β-strands [77], consistent with the characterization of GOF and LOF variants established by other features. GOF variants were also enriched in proteins capable of forming homomultimers suggesting a potential dominant negative pattern of gain of function for some of the variants and further emphasizing the necessity to investigate protein interactions when assessing variant functional impact (Additional file 2: Fig. S3). Interestingly, we found GOF and LOF variants to have a greater number of pathogenic variants in neighboring residues than do neutral variants in our dataset. Together, these observations indicate significant divergence between GOF and LOF variants in their mode of pathogenicity at the protein level and suggest several mechanisms that may guide and inform the investigation of individual variants. Further, these results demonstrate that AF2-predicted protein structures may provide significant biological signal in variant assessment tasks and can facilitate the extraction of protein structural features proteome-wide.

Previous studies have also attempted to establish and investigate databases of experimentally identified GOF and LOF variants. One such study identified 258 LOF variants and 129 GOF variants in 168 genes and suggested that LOF variants appear to impact protein function more severely than GOF variants, similar to our findings in this work [76]. However, the variants identified in that study were not selected to include only pathogenic variants, and, further, GOF variants that result in increased protein function were omitted from that work. Heyne et al. [9] examined 518 LOF variants and 309 GOF variants in 12 SCNxA and CACNA1x family genes, labeled on the basis of known gene-disease mechanisms. The authors identified several features relating to protein structure as well as qualities specific to transmembrane proteins by which the distributions for GOF and LOF variants differed significantly and employed those features to develop a classifier for variants in the SCNxA/CACNA1x genes. In a recent study, GOF and LOF labels were inferred for pathogenic variants based on the suspected mode of inheritance for gene-related disorders, resulting in a total of 7357 putative LOF and 2877 putative non-LOF variants (GOF + dominant negative). Similarly to our analysis, the authors compare predicted protein stability changes between GOF and LOF variants to indicate that LOF variants are more disruptive to protein structures [75]. Further, the authors suggest that GOF variants tend to cluster more closely in 3D space than do LOF variants. Conversely, we do not observe GOF variants to be surrounded by a significantly greater number of pathogenic variants than LOF variants (Additional File 2: Fig. S3). While these findings are seemingly at odds, the study in question considers only known GOF or LOF variants when examining clustering proclivity, whereas we consider all known pathogenic variants regardless of GOF or LOF status, possibly accounting for this discrepancy.

Second, LoGoFunc demonstrates strong performance on a test set of GOF, LOF, and neutral variants and achieves substantial improvement over the baseline in its classification of the functional impact of genetic variants, addressing a long-standing need for high-throughput methods that discriminate between these important mechanisms of variant pathogenicity (Fig. 3). This is likewise notable considering the benchmarked tools in our analysis generally performed better on LOF variants than GOF, as has been similarly observed in previous studies [14, 75]. Particularly, many pathogenicity predictors such as CADD [4] and REVEL [7] tend to predict LOF variants as pathogenic or deleterious more often than GOF variants, whereas GOF variants are more often predicted to be benign [14, 75]. This may be due in part to the under-representation of GOF variants in the training data used by these tools where applicable or may arise because GOF variants may be difficult to separate from neutral variants using the features or methods employed by these tools. Importantly, LoGoFunc considers pathogenic variant functional effects during training and includes features selected to identify pathogenic variants with mechanisms beyond protein destabilization, allowing it to provide state-of-the-art performance for the discrimination of pathogenic GOF and LOF variants.

Third, our analysis identified previously undocumented associations between various biological features and the functional outcomes of genetic variants. We assessed the importance of the features used to train LoGoFunc and found that the model learns from a diverse array of gene-, protein-, and variant-level features including functional, conservation, structural, and network information (Fig. 4). For example, we processed the STRING [39] PPI network using node2vec [40] to summarize the human protein interactome. Whereas some models have included binary indications of the involvement of a protein in any protein interaction [20], to our knowledge, such PPI network features are rarely used in popular pathogenicity prediction methods. Yet, many dimensions of the output are highly impactful for the LoGoFunc model, suggesting protein function at the pathway- and/or systems-level may have a bearing on variant pathogenicity and functional effect. Concordantly, PPI features are accompanied by several other protein sequence- and structure-based features from which the model also learns, including top features such as DDGun [36] stability impact predictions, residue proximal pathogenic variants, and the AF2 structure pLDDT values which have been shown to correlate significantly with protein structural disorder [12]. Genic context also has a substantial impact on the model’s output as evidenced by the inclusion of several gene-level features such as the gene damage index [78] and the number of gene paralogs. Other important features, such as the per variant predictions of pathogenicity for autosomal dominant or recessive disease, align with previous characterizations of GOF and LOF variants, thereby supporting the biological plausibility of LoGoFunc’s predictions and lending credence to the novel associations we identified between various features employed by the model and GOF, LOF, and neutral variants.

Finally, we illustrate LoGoFunc’s potential utility in identifying clinically relevant variants. Although our PheWAS approach relies on EHR data, which lacks granularity for rare diseases where most disease-causing variants typically contribute and variant-level association testing usually demands a significantly large sample size, we have identified strong associations between the predicted GOF and LOF variants and relevant phenotypes using robust association testing methods. Our results both corroborate previously known associations of GOF and LOF effects and aid in the identification of novel plausible candidates. For example, we found that two predicted LOF variants in *HBB* p.Glu7Lys and p.Glu27Lys were associated with hereditary hemolytic anemias, while a predicted neutral variant demonstrated a reduced risk. While p.Glu27Lys has been reported as a pathogenic variant, p.Glu7Lys has conflicting interpretations of pathogenicity in ClinVar [16]. Our results suggest that the p.Glu7Lys variant may indeed be pathogenic. Additionally, among the three variants in *TTR* that were previously classified as benign/likely benign or variant of uncertain significance (VUS), two were predicted as LOF by LoGoFunc, while one was predicted as neutral. *TTR* encodes transthyretin, and pathogenic forms of transthyretin are known to cause hereditary amyloidosis [79]. In line with LoGoFunc's predictions, the two variants predicted as LOF were associated with amyloidosis, whereas the predicted neutral variant was not.

Furthermore, of the three predicted LOF variants in *HFE*, which have conflicting interpretations in ClinVar, two were associated with disorders of iron metabolism in BioMe BioBank. Pathogenic variants in *HFE* are recognized as the cause of autosomal recessive hemochromatosis, a disorder of iron metabolism [80]. The predicted LOF variant that failed to display an association with disorders of iron metabolism was only observed in a heterozygous state, possibly preventing the manifestation of a phenotypic effect. Moreover, we observed two variants in *PSEN1*, one predicted as LOF (p.Gly206Ala) and one predicted as neutral (p.Glu318Gly). *PSEN1* is linked to frontotemporal dementia and Alzheimer’s disease [81]. LoGoFunc's predictions aligned with previously reported associations in ClinVar, with p.Gly206Ala associated with the phecode for dementias while p.Glu318Gly was not associated. Another predicted GOF variant, p.Asn2081Asp in *LRRK2*, reported as benign in ClinVar, but has been previously shown to have a GOF effect and associated with Crohn’s disease [82]. Consistently, LoGoFunc identified this variant as a predicted GOF, and our PheWAS analysis confirmed its association with regional enteritis in BioMe. Other examples include a predicted GOF variant in *PTPN22*, p.Trp620Arg, associated with type 1 diabetes. p.Trp620Arg has been previously reported as a GOF variant altering T cell response and linked to autoimmune diseases, including type 1 diabetes mellitus [83]. Lastly, LoGoFunc predicted a GOF variant in *JAK2*, p.Val617Phe, which has been hypothesized to confer a proliferative advantage in hematopoietic precursor cells [84] and was associated with myeloproliferative disease in BioMe BioBank. Together, these results provide preliminary evidence that LoGoFunc may provide utility for identifying clinically relevant variants and in the assessment of VUS and uncharacterized variants in addition to providing predictions of functional effect.

## Conclusions

In summary, we have developed LoGoFunc, a predictor of GOF, LOF, and neutral variants. Our model performs favorably compared to commonly used computational tools designed for the assessment of genetic variation and demonstrates strong predictive power across metrics on our test set of GOF, LOF, and neutral variants. We assessed the contribution of various features to the model’s output and found that LoGoFunc learns from a diverse array of structural, functional, sequence-based, and systems-level information, indicating that these features have a bearing on the functional outcome of genetic variants. Further, we demonstrated significant localization of GOF, LOF, and neutral variants in 3D structural and functional sites in proteins, and demonstrated LoGoFunc’s ability to assess previously uncharacterized variants. Our findings corroborated previously reported molecular mechanisms resulting in the gain or loss of function and also suggest novel mechanisms that may shed light on disease etiology. We applied our method to 82,468,698 canonical missense mutations in the human genome and provide our predictions, which are freely available to noncommercial users, at https://itanlab.shinyapps.io/goflof/ [13].

### Supplementary Information

**Additional file 1: Table S1.** Number of variants per class in complete, training, and testing datasets. **Table S2.** Description of features used for LoGoFunc’s development. **Table S3.** Fisher's exact test results comparing the GOF, LOF, and neutral variants across protein features. **Table S4.** Odds ratios and log odds ratios comparing the GOF, LOF, and neutral variants across protein features. **Table S5.** Fisher's exact test results comparing the GOF and LOF variants across protein features. **Table S6.** Odds ratios and log odds ratios comparing the GOF and LOF variants across protein features. **Table S7.** GOF, LOF, and neutral prediction 95% confidence intervals per gene. **Table S8.** Feature importance values for features used to train LoGoFunc. **Table S9.** PheWAS using predicted GOF and LOF missense variants in the BioMe BioBank cohort. **Table S10.** Replicated PheWas results on an independent BioMe WES cohort.

**Additional file 2: Supplementary information Fig. S1.** Nested cross-validation strategy employed for model selection. **Fig. S2.** Box and whisker plots for the comparison of tested model architecture performance on the outer folds of the nested cross-validation loop. **Fig. S3.** Enrichments and depletions for protein structural and functional features used by the LoGoFunc model. **Fig. S4.** Precision-recall curves by variant class for all variants in the homology-filtered test set. **Fig. S5.** Precision-recall curves indicating the discriminatory power of various pathogenicity prediction methods and LoGoFunc on a set of variants from ClinVar. **Fig. S6.** Precision-recall curves indicating the discriminatory power of various pathogenicity prediction methods and LoGoFunc on variants from the test set. **Fig. S7.** Precision-recall curves comparing the discriminatory power of various pathogenicity prediction methods and LoGoFunc on a set of variants from the homology-filtered test set. **Fig. S8.** Precision-recall curves comparing the discriminatory power of funNCion and LoGoFunc on variants from the funNCion testing dataset. **Fig. S9.** Precision-recall curves comparing the discriminatory power of VPatho and LoGoFunc on a set of variants from the test set for which predictions were available from both tools. **Fig. S10.** Precision-recall curves indicating the discriminatory power of mode of inheritance predictions from MOI-pred and LoGoFunc. **Fig. S11.** Comparison of splicing-related features for GOF, LOF, and neutral variants.

## Data Availability

Functional predictions generated with LoGoFunc are available at https://itanlab.shinyapps.io/goflof/ and https://zenodo.org/records/10126185 [13]. Data used for training and testing LoGoFunc is available at https://gitlab.com/itan-lab/logofunc and https://zenodo.org/record/7916161 [85]. Annotation data for missense variants are available at https://zenodo.org/record/7562029 [86]. Code to train and test LoGoFunc is available at https://gitlab.com/itan-lab/logofunc and https://zenodo.org/record/7916161 [85]. A user-friendly website for querying precomputed LoGoFunc predictions is available at https://itanlab.shinyapps.io/goflof/.

## Abbreviations

GOF: Gain-of-function
LOF: Loss-of-function
CMC: Chronic mucocutaneous candidiasis
MSMD: Mendelian Susceptibility to Mycobacterial Disease
AF2: AlphaFold2
HGMD: Human Gene Mutation Database
DM: Disease-causing class variants
NLP: Natural language processing
VEP: Variant effect predictor
RSA: Relative solvent accessibility
F1: Macro-averaged F1-score
AP: Average precision
WES: Whole exome sequencing
EHR: Electronic health records
PCA: Principal component analysis
LD: Linkage disequilibrium
ln(OR): Natural logarithm of odds ratio
PPI: Protein–protein interaction
pLDDT: Predicted local distance difference test
CSS: Cryptic splice sites
MCC: Matthew’s Correlation Coefficient
OR: Odds ratio
AD: Autosomal dominant
AR: Autosomal recessive
PDB: Protein Data Bank
PheWAS: Phenome-wide association study
VUS: Variant of uncertain or conflicting significance
PTM: Post-translationally modified residue

## References

[1] Studer RA, Dessailly BH, Orengo CA (2013). Residue mutations and their impact on protein structure and function: detecting beneficial and pathogenic changes. Biochem J.

[2] Boisson-Dupuis S, Kong X-F, Okada S, Cypowyj S, Puel A, Abel L (2012). Inborn errors of human STAT1: allelic heterogeneity governs the diversity of immunological and infectious phenotypes. Curr Opin Immunol.

[3] Gupta K, Varadarajan R (2018). Insights into protein structure, stability and function from saturation mutagenesis. Curr Opin Struct Biol.

[4] Kircher M, Witten DM, Jain P, O’Roak BJ, Cooper GM, Shendure J (2014). A general framework for estimating the relative pathogenicity of human genetic variants. Nat Genet.

[5] Adzhubei IA, Schmidt S, Peshkin L, Ramensky VE, Gerasimova A, Bork P (2010). A method and server for predicting damaging missense mutations. Nat Methods.

[6] Ng PC, Henikoff S (2003). SIFT: predicting amino acid changes that affect protein function. Nucleic Acids Res.

[7] Ioannidis NM, Rothstein JH, Pejaver V, Middha S, McDonnell SK, Baheti S (2016). REVEL: an ensemble method for predicting the pathogenicity of rare missense variants. Am J Hum Genet.

[8] Feng B-J (2017). PERCH: a unified framework for disease gene prioritization. Hum Mutat.

[9] Heyne HO, Baez-Nieto D, Iqbal S, Palmer DS, Brunklaus A, May P (2020). Predicting functional effects of missense variants in voltage-gated sodium and calcium channels. Sci Transl Med.

[10] Ge F, Li C, Iqbal S, Muhammad A, Li F, Thafar MA (2023). VPatho: a deep learning-based two-stage approach for accurate prediction of gain-of-function and loss-of-function variants. Brief Bioinform.

[11] Ke G, Meng Q, Finley T, Wang T, Chen W, Ma W, et al. LightGBM: a highly efficient gradient boosting decision tree. Proc 31st Int Conf Neural Inf Process Syst. 2017;30:3149–57. Red Hook: Curran Associates Inc.

[12] Jumper J, Evans R, Pritzel A, Green T, Figurnov M, Ronneberger O (2021). Highly accurate protein structure prediction with AlphaFold. Nature.

[13] Stein D, Ece Kars M, Wu Y, Sevim Bayrak C, Stenson PD, Cooper DN, et al. LoGoFunc predictions. Zenodo. 2023. 10.5281/zenodo.10126185.

[14] Sevim Bayrak C, Stein D, Jain A, Chaudhary K, Nadkarni GN, Van Vleck TT (2021). Identification of discriminative gene-level and protein-level features associated with pathogenic gain-of-function and loss-of-function variants. Am J Hum Genet.

[15] Stenson PD, Mort M, Ball EV, Evans K, Hayden M, Heywood S (2017). The human gene mutation database: towards a comprehensive repository of inherited mutation data for medical research, genetic diagnosis and next-generation sequencing studies. Hum Genet.

[16] Landrum MJ, Lee JM, Benson M, Brown GR, Chao C, Chitipiralla S (2018). ClinVar: improving access to variant interpretations and supporting evidence. Nucleic Acids Res.

[17] Karczewski KJ, Francioli LC, Tiao G, Cummings BB, Alföldi J, Wang Q (2020). The mutational constraint spectrum quantified from variation in 141,456 humans. Nature.

[18] Pearson WR. An introduction to sequence similarity (“homology”) searching. Curr Protoc Bioinforma Ed Board Andreas Baxevanis Al. 2013;03. 10.1002/0471250953.bi0301s42. 10.1002/0471250953.bi0301s42 PMC3820096 23749753

[19] Fu L, Niu B, Zhu Z, Wu S, Li W (2012). CD-HIT: accelerated for clustering the next-generation sequencing data. Bioinforma Oxf Engl.

[20] Pejaver V, Urresti J, Lugo-Martinez J, Pagel KA, Lin GN, Nam H-J (2020). Inferring the molecular and phenotypic impact of amino acid variants with MutPred2. Nat Commun.

[21] McLaren W, Gil L, Hunt SE, Riat HS, Ritchie GRS, Thormann A (2016). The Ensembl Variant Effect Predictor. Genome Biol.

[22] González-Pérez A, López-Bigas N (2011). Improving the assessment of the outcome of nonsynonymous SNVs with a consensus deleteriousness score. Condel Am J Hum Genet.

[23] Liu X, Li C, Mou C, Dong Y, Tu Y (2020). dbNSFP v4: a comprehensive database of transcript-specific functional predictions and annotations for human nonsynonymous and splice-site SNVs. Genome Med.

[24] Siepel A, Bejerano G, Pedersen JS, Hinrichs AS, Hou M, Rosenbloom K (2005). Evolutionarily conserved elements in vertebrate, insect, worm, and yeast genomes. Genome Res.

[25] Garber M, Guttman M, Clamp M, Zody MC, Friedman N, Xie X (2009). Identifying novel constrained elements by exploiting biased substitution patterns. Bioinformatics.

[26] Davydov EV, Goode DL, Sirota M, Cooper GM, Sidow A, Batzoglou S (2010). Identifying a high fraction of the human genome to be under selective constraint using GERP++. PLoS Comput Biol.

[27] Henikoff S, Henikoff JG (1992). Amino acid substitution matrices from protein blocks. Proc Natl Acad Sci U S A.

[28] Cooper GM, Stone EA, Asimenos G, Green ED, Batzoglou S, NISC Comparative Sequencing Program (2005). Distribution and intensity of constraint in mammalian genomic sequence. Genome Res.

[29] Shamsani J, Kazakoff SH, Armean IM, McLaren W, Parsons MT, Thompson BA (2019). A plugin for the ensembl variant effect predictor that uses MaxEntScan to predict variant spliceogenicity. Bioinformatics.

[30] Jian X, Boerwinkle E, Liu X (2014). In silico prediction of splice-altering single nucleotide variants in the human genome. Nucleic Acids Res.

[31] Varadi M, Anyango S, Deshpande M, Nair S, Natassia C, Yordanova G (2022). AlphaFold protein structure database: massively expanding the structural coverage of protein-sequence space with high-accuracy models. Nucleic Acids Res.

[32] Berman HM, Westbrook J, Feng Z, Gilliland G, Bhat TN, Weissig H (2000). The Protein Data Bank. Nucleic Acids Res.

[33] UniProt Consortium (2021). UniProt: the universal protein knowledgebase in 2021. Nucleic Acids Res.

[34] Kabsch W, Sander C (1983). Dictionary of protein secondary structure: pattern recognition of hydrogen-bonded and geometrical features. Biopolymers.

[35] Predicting Protein Ligand Binding Sites by Combining Evolutionary Sequence Conservation and 3D Structure | PLOS Computational Biology. Available from: https://journals.plos.org/ploscompbiol/article?id=, 10.1371/journal.pcbi.1000585. Cited 20 Oct 2022. 10.1371/journal.pcbi.1000585 PMC2777313 19997483

[36] Montanucci L, Capriotti E, Frank Y, Ben-Tal N, Fariselli P (2019). DDGun: an untrained method for the prediction of protein stability changes upon single and multiple point variations. BMC Bioinformatics.

[37] Xia Y, Xia C-Q, Pan X, Shen H-B (2021). GraphBind: protein structural context embedded rules learned by hierarchical graph neural networks for recognizing nucleic-acid-binding residues. Nucleic Acids Res.

[38] Wilson CJ, Choy W-Y, Karttunen M (2022). AlphaFold2: a role for disordered protein/region prediction?. Int J Mol Sci.

[39] Szklarczyk D, Gable AL, Lyon D, Junge A, Wyder S, Huerta-Cepas J (2019). STRING v11: protein–protein association networks with increased coverage, supporting functional discovery in genome-wide experimental datasets. Nucleic Acids Res.

[40] Grover A, Leskovec J. node2vec: scalable feature learning for networks. ArXiv160700653 Cs Stat. 2016; Available from: http://arxiv.org/abs/1607.00653. Cited 30 Mar 2022. 10.1145/2939672.2939754 PMC5108654 27853626

[41] Zhao B, Katuwawala A, Oldfield CJ, Dunker AK, Faraggi E, Gsponer J (2021). DescribePROT: database of amino acid-level protein structure and function predictions. Nucleic Acids Res.

[42] Grantham R (1974). Amino acid difference formula to help explain protein evolution. Science.

[43] Cheng J, Nguyen TYD, Cygan KJ, Çelik MH, Fairbrother WG, Avsec Ž (2019). MMSplice: modular modeling improves the predictions of genetic variant effects on splicing. Genome Biol.

[44] Benjamini Y, Hochberg Y (1995). Controlling the false discovery rate: a practical and powerful approach to multiple testing. J R Stat Soc Ser B Methodol.

[45] Lundberg SM, Lee S-I. A unified approach to interpreting model predictions. Adv Neural Inf Process Syst. Curran Associates, Inc.; 2017. Available from: https://proceedings.neurips.cc/paper/2017/hash/8a20a8621978632d76c43dfd28b67767-Abstract.html. Cited 22 Jun 2022.

[46] Breiman L (2001). Random forests. Mach Learn.

[47] Chen T, Guestrin C. XGBoost: a scalable tree boosting system. Proc 22nd ACM SIGKDD Int Conf Knowl Discov Data Min. 2016;785–94.

[48] Akiba T, Sano S, Yanase T, Ohta T, Koyama M. Optuna: a next-generation hyperparameter optimization framework. ArXiv190710902 Cs Stat. 2019; Available from: http://arxiv.org/abs/1907.10902. Cited 30 Mar 2022.

[49] Kotsiantis SB (2013). Decision trees: a recent overview. Artif Intell Rev.

[50] Massey FJ (1951). The Kolmogorov-Smirnov test for goodness of fit. J Am Stat Assoc.

[51] Virtanen P, Gommers R, Oliphant TE, Haberland M, Reddy T, Cournapeau D (2020). SciPy 1.0: fundamental algorithms for scientific computing in Python. Nat Methods.

[52] Mapping ICD-10 and ICD-10-CM Codes to Phecodes: Workflow development and initial evaluation - PubMed. Available from: https://pubmed-ncbi-nlm-nih-gov.eresources.mssm.edu/31553307/. Cited 18 Sep 2023. 10.2196/14325 PMC6911227 31553307

[53] Purcell S, Neale B, Todd-Brown K, Thomas L, Ferreira MAR, Bender D (2007). PLINK: a tool set for whole-genome association and population-based linkage analyses. Am J Hum Genet.

[54] Mbatchou J, Barnard L, Backman J, Marcketta A, Kosmicki JA, Ziyatdinov A (2021). Computationally efficient whole-genome regression for quantitative and binary traits. Nat Genet.

[55] Ma C, Blackwell T, Boehnke M, Scott LJ, GoT2D investigators (2013). Recommended joint and meta-analysis strategies for case-control association testing of single low-count variants. Genet Epidemiol.

[56] Steinegger M, Söding J (2017). MMseqs2 enables sensitive protein sequence searching for the analysis of massive data sets. Nat Biotechnol.

[57] Mistry J, Chuguransky S, Williams L, Qureshi M, Salazar GA, Sonnhammer ELL (2021). Pfam: the protein families database in 2021. Nucleic Acids Res.

[58] Blum M, Chang H-Y, Chuguransky S, Grego T, Kandasaamy S, Mitchell A (2021). The InterPro protein families and domains database: 20 years on. Nucleic Acids Res.

[59] Quang D, Chen Y, Xie X (2015). DANN: a deep learning approach for annotating the pathogenicity of genetic variants. Bioinforma Oxf Engl.

[60] Alirezaie N, Kernohan KD, Hartley T, Majewski J, Hocking TD (2018). ClinPred: prediction tool to identify disease-relevant nonsynonymous single-nucleotide variants. Am J Hum Genet.

[61] Lu Q, Hu Y, Sun J, Cheng Y, Cheung K-H, Zhao H (2015). A statistical framework to predict functional non-coding regions in the human genome through integrated analysis of annotation data. Sci Rep.

[62] Liu X, Wu C, Li C, Boerwinkle E (2016). dbNSFP v3.0: a one-stop database of functional predictions and annotations for human non-synonymous and splice site SNVs. Hum Mutat.

[63] Sundaram L, Gao H, Padigepati SR, McRae JF, Li Y, Kosmicki JA (2018). Predicting the clinical impact of human mutation with deep neural networks. Nat Genet.

[64] Pan X, Li Z, Huang X, Huang G, Gao S, Shen H (2019). Molecular basis for pore blockade of human Na+ channel Nav1.2 by the μ-conotoxin KIIIA. Science.

[65] Eilbeck K, Lewis SE, Mungall CJ, Yandell M, Stein L, Durbin R (2005). The sequence ontology: a tool for the unification of genome annotations. Genome Biol.

[66] Carter H, Douville C, Stenson PD, Cooper DN, Karchin R (2013). Identifying Mendelian disease genes with the variant effect scoring tool. BMC Genomics.

[67] Jagadeesh KA, Wenger AM, Berger MJ, Guturu H, Stenson PD, Cooper DN (2016). M-CAP eliminates a majority of variants of uncertain significance in clinical exomes at high sensitivity. Nat Genet.

[68] Qi H, Zhang H, Zhao Y, Chen C, Long JJ, Chung WK (2021). MVP predicts the pathogenicity of missense variants by deep learning. Nat Commun.

[69] Petrazzini BO, Balick DJ, Forrest IS, Cho J, Rocheleau G, Jordan DM, et al. Prediction of recessive inheritance for missense variants in human disease. MedRxiv; 2021. 2021.10.25.21265472. Available from: https://www.medrxiv.org/content/, 10.1101/2021.10.25.21265472v1. Cited 30 Mar 2022.

[70] Samocha KE, Robinson EB, Sanders SJ, Stevens C, Sabo A, McGrath LM (2014). A framework for the interpretation of de novo mutation in human disease. Nat Genet.

[71] Itan Y, Shang L, Boisson B, Ciancanelli MJ, Markle JG, Martinez-Barricarte R (2016). The mutation significance cutoff: gene-level thresholds for variant predictions. Nat Methods.

[72] Khurana E, Fu Y, Chen J, Gerstein M (2013). Interpretation of genomic variants using a unified biological network approach. PLOS Comput Biol.

[73] Reynolds C, King MD, Gorman KM (2020). The phenotypic spectrum of SCN2A-related epilepsy. Eur J Paediatr Neurol EJPN Off J Eur Paediatr Neurol Soc.

[74] Spratt PWE, Ben-Shalom R, Keeshen CM, Burke KJ, Clarkson RL, Sanders SJ (2019). The autism-associated gene Scn2a contributes to dendritic excitability and synaptic function in the prefrontal cortex. Neuron.

[75] Gerasimavicius L, Livesey BJ, Marsh JA (2022). Loss-of-function, gain-of-function and dominant-negative mutations have profoundly different effects on protein structure. Nat Commun.

[76] Jung S, Lee S, Kim S, Nam H (2015). Identification of genomic features in the classification of loss- and gain-of-function mutation. BMC Med Inform Decis Mak.

[77] Abrusán G, Marsh JA (2016). Alpha helices are more robust to mutations than beta strands. PLoS Comput Biol.

[78] Itan Y, Shang L, Boisson B, Patin E, Bolze A, Moncada-Vélez M (2015). The human gene damage index as a gene-level approach to prioritizing exome variants. Proc Natl Acad Sci U S A.

[79] Ando Y, Nakamura M, Araki S (2005). Transthyretin-related familial amyloidotic polyneuropathy. Arch Neurol.

[80] Allen KJ, Gurrin LC, Constantine CC, Osborne NJ, Delatycki MB, Nicoll AJ (2008). Iron-overload-related disease in HFE hereditary hemochromatosis. N Engl J Med.

[81] Bagaria J, Bagyinszky E, An SSA (2022). Genetics, functions, and clinical impact of Presenilin-1 (PSEN1) gene. Int J Mol Sci.

[82] Hui KY, Fernandez-Hernandez H, Hu J, Schaffner A, Pankratz N, Hsu N-Y (2018). Functional variants in the LRRK2 gene confer shared effects on risk for Crohn’s disease and Parkinson’s disease. Sci Transl Med.

[83] Cao Y, Yang J, Colby K, Hogan SL, Hu Y, Jennette CE (2012). High basal activity of the PTPN22 gain-of-function variant blunts leukocyte responsiveness negatively affecting IL-10 production in ANCA vasculitis. PLoS ONE.

[84] Kralovics R, Passamonti F, Buser AS, Teo S-S, Tiedt R, Passweg JR (2005). A gain-of-function mutation of JAK2 in myeloproliferative disorders. N Engl J Med.

[85] Stein D, Ece Kars M, Wu Y, Sevim Bayrak C, Stenson PD, Cooper DN, et al. LoGoFunc model. Zenodo. 2023. 10.5281/zenodo.7916161 .

[86] Stein D, Ece Kars M, Wu Y, Sevim Bayrak C, Stenson PD, Cooper DN, et al. Annotated missense variants (hg38) for LoGoFunc prediction. Zenodo. 2022. 10.5281/zenodo.7562029 .

