# Supplementary material for: Genome-wide prediction of pathogenic gain- and loss-of-function variants from ensemble learning of a diverse feature set
Source: Genome Med. 2023 Nov 30;15:103. doi: 10.1186/s13073-023-01261-9 (PMC10688473; doi:10.1186/s13073-023-01261-9)
Supplement: Supplementary file 2 — Additional file 2: Supplementary information Fig. S1. Nested cross-validation strategy employed for model selection. Fig. S2. Box and whisker plots for the comparison of tested model architecture performance on the outer folds of the nested cross-validation loop. Fig. S3. Enrichments and depletions for protein structural and functional features used by the LoGoFunc model. Fig. S4. Precision-recall curves by variant class for all variants in the homology-filtered test set. Fig. S5. Precision-recall curves indicating the discriminatory power of various pathogenicity prediction methods and LoGoFunc on a set of variants from ClinVar. Fig. S6. Precision-recall curves indicating the discriminatory power of various pathogenicity prediction methods and LoGoFunc on variants from the test set. Fig. S7. Precision-recall curves comparing the discriminatory power of various pathogenicity prediction methods and LoGoFunc on a set of variants from the homology-filtered test set. Fig. S8. Precision-recall curves comparing the discriminatory power of funNCion and LoGoFunc on variants from the funNCion testing dataset. Fig. S9. Precision-recall curves comparing the discriminatory power of VPatho and LoGoFunc on a set of variants from the test set for which predictions were available from both tools. Fig. S10. Precision-recall curves indicating the discriminatory power of mode of inheritance predictions from MOI-pred and LoGoFunc. Fig. S11. Comparison of splicing-related features for GOF, LOF, and neutral variants. [file 13073_2023_1261_MOESM2_ESM.docx]

**Supplementary methods**

**Annotation**

*Conservation*. Primate, mammal, and vertebrate PhastCons [1], and PhyloP [1] scores excluding *Homo sapiens*, were obtained as precalculated annotations provided by CADD v1.6 [2] for all human missense mutations in the GRCh38 reference genome and selected indels from gnomAD [3]. PhastCons and PhyloP scores including *Homo sapiens* in the alignments were obtained from dbNSFP [4] via VEP [5]. Grantham [6], Ex [7], PAM250 [8], JM [9], and VB [10] substitution scores were obtained from a SQL database of protein annotations provided by SNVBox [11]. dbNSFP was queried via VEP to obtain GERP++ [12] and SiPhy_29way [13] conservation scores. PSIC [14] scores for wild-type and mutant amino acids and the number of observed amino acids at the substitution position were obtained as precomputed annotations provided by PolyPhen-2 [15]. GERP [16] conservation scores were procured from VEP and the CADD v1.6 database. Finally, conservation metrics based on MMSeq2 [17] alignments were queried from the DescribePROT [18] database for the human proteome.

*Gene and protein sequence.* Percent GC and CpG in +/−75 base-pair windows around the mutation sites were obtained from the CADD v1.6 annotation database. cDNA positions, coding sequence positions, protein positions, coding sequence strands, and nucleotide positions in the codon were obtained with VEP. VEP was also used to determine the distance from the nearest exon junction boundary within 10,000 base pairs and the length of the nearest exon. Distances to the nearest transcription start sites and transcribed sequence ends were obtained from CADD v1.6.

*Genomic motifs, domains, states, and functional regions.* The number of overlapping motifs, highly informative motif positions, and motif score changes when transitioning from wild-type to mutant allele were obtained from the CADD v1.6 annotation database. Proximity to splice sites and the nearest mutation in BRAVO were also collected from CADD. Finally, overlapping regulatory features, transcription factor binding sites, chromatin states, and miRNA target predictions were obtained from the CADD v1.6 annotation database.

*Allele Frequencies and Mutational Occurrence.* Allele frequencies from the 1000 Genomes Project [19] and the UK10K TWINSUK cohort [20] were collected via the dbNSFP plugin for VEP. The number of frequent (MAF > 0.05), rare (MAF < 0.05), and single occurrence SNVs in BRAVO in 100 and 1000 base-pair windows were obtained from CADD. The frequencies of missense substitution types from COSMIC [21] and HapMap [22] as well as the frequency of missense substitution types in COSMIC normalized by frequency of the reference residue in human proteins in SwissProt/TrEMBL [23] and normalized by the number of times the substitution type was identified in HapMap were queried from SNVBox.

*Protein Structure and Composition*. Predictions of protein accessible surface area, disordered flexible linker residues, disordered RNA, DNA, and protein binding residues, RNA and DNA binding residues, MoRF regions, protein secondary structures, protein binding residues, signal peptides, and intrinsically disordered residues, were obtained from the DescribePROT database for the human proteome. Post-translational modifications were collected from iPTMnet [24] and dbPTM [25]. NetSurfP version 1.0d [26] was used to calculate additional estimates of relative and total accessible surface area along with reliability estimates for those scores. We calculated additional predictions of disordered protein regions and disordered binding regions with IUPred2A [27] and ANCHOR2 [27], respectively. Pfam [28] and Interpro protein domains were queried from the Ensembl BioMart [30]. Changes in residue side chain volume and solvent accessible surface area, normalized B-factor for the residue, number of hydrogen sidechain-sidechain and sidechain-mainchain bonds formed by the residue, and the average number of residue contacts with heteroatoms per homologous PDB chain along with the closest residue contact with a heteroatom were obtained from the PolyPhen-2 annotation database. The average number of residue contacts with other chains per homologous PDB chain, the closest residue contact with another chain, the average number of residue contacts with critical sites per homologous PDB chain, and the closest residue contact with a critical site were also collected from PolyPhen-2. Documented modified residues and regions of interest were obtained from UniProt.

*Genic characterizations*. Predicted and experimentally derived haploinsufficient genes, mode of inheritance predictions, gene selective pressure estimates, genic tolerance to variation, and gene damage metrics were obtained from their respective web servers (Supplementary Table 1). Numbers of paralogs per gene were queried from Ensembl BioMart.

*Pathogenicity and deleteriousness.* SIFT4G [31], BayesDel [32], ClinPred [33], DANN [34], DEOGEN2 [35], Eigen [36], FATHMM [37], LINSIGHT [38], LIST_S2 [39], LRT [40], M_CAP [41], MPC [42], MVP [43], MetaLR [4], MetaSVM [4], MutPred [44], MutationAssessor [45], MutationTaster 2 [46], PROVEAN [47], PrimateAI [48], VEST4 [49], MSC [50], and fitCons [51] estimates of pathogenicity and deleteriousness were obtained with VEP and the dbNSFP plugin. Variant consequences and their relative impact were also retrieved from VEP. CADD [52], PolyPhen-2, SIFT [53], and CONDEL [54] scores were obtained with VEP.

*Splicing.* SpliceAI [55] and MMSplice [56] scores were obtained from the CADD v1.6 annotation database along with dbscSNV [57] Adaboost and RandomForest classifier scores. MaxEntScan [58] scores were collected via the eponymous VEP plugin.

*Expression and epigenetics.* ENCODE [59] histone modification levels, chromatin state characterizations, and overlapping transcription factor binding sites and regulatory features were obtained from CADD v1.6. Median transcripts per million across 54 tissue types were obtained from GTEx.

*Protein-protein interactions.* The protein-protein interaction network defined across evidence channels from the STRING [60] database version 11 was dimensionally reduced to 64 features with the node2vec [61] implementation found at [https://github.com/eliorc/node2vec](about:blank) with default parameters.

**GOF, LOF, and neutral variant effects on splicing**

Splice-disrupting variants have been reported to constitute the second largest class of known disease-causing mutations, and have been found to yield both GOF and LOF phenotypes [55,56]. Given the importance of splice disruption as a general causal disease mechanism, we investigated the distribution of splicing-related features among the classes. Notably, LOF variants were located most closely to splice sites followed by GOF and neutral variants, respectively (p-values 2.36E-09, 1.12E-08) (Supplementary Figure 9a, 9c). Further, LOF variants were significantly enriched for the loss of cryptic splice acceptor and donor sites (p-values 9.28E-04, 2.52E-07) - potentially important mechanisms of alternative splicing – and significantly depleted for the gain of cryptic splice acceptor and donor sites (p-values 2.74E-08, 1.55E-15) (Supplementary Figure 9a). By contrast, neutral variants were enriched for the gain of splice acceptor and donor sites. The enrichment of neutral variants for the gain of cryptic splice sites (CSS) is potentially explicable in terms of the ability of canonical splice sites to suppress CSS activation [62]. Thus, these CSSs acquired via neutral mutations may not have a significant impact on transcript expression. After removing variants not predicted to impact splicing, LOF variants were predicted to lead to a greater decrease in the proportion spliced-in (Ψ) than GOF or neutral variants based on estimates from the MMSplice [56] exon, donor, and acceptor predictors (Supplementary Figure 9b). LOF variants were similarly predicted to lead to a greater decrease in Ψ than neutral variants based on the donor-intron and acceptor-intron MMSplice predictions. GOF variants lead to a greater decrease in Ψ than neutral variants based on the exon and donor predictions (Supplementary Figure 9b). These results indicate that LOF variants in particular, and to a lesser extent GOF variants, may exert their pathogenic effects via the disruption of canonical splicing patterns.

**Hyperparameter tuning**

Hyperparameters were tuned using the Optuna [63] optimization library version 2.10.0 with the Tree-structured Parzen Estimator to sample from the hyperparameter search spaces. We used the LightGBM [64] implementation version 3.2.1 from [https://github.com/microsoft/LightGBM](about:blank), and the scikit-learn [65] API of the XGBoost [66] implementation version 1.5.0 from [https://github.com/dmlc/xgboost](about:blank). We used the RandomForest [67] implementation from scikit-learn library version 1.1.1. Neural networks were implemented using Pytorch [68] version 1.8.0. For LightGBM, XGBoost, and RandomForest, parameters in the search spaces below correspond to the parameters defined in the documentations of the implementations located at [https://lightgbm.readthedocs.io/en/latest/Parameters.html](about:blank), [https://github.com/dmlc/xgboost/blob/master/doc/parameter.rst](about:blank), and [https://scikit-learn.org/stable/modules/generated/sklearn.ensemble.RandomForestClassifier.html](about:blank) respectively. For the neural network implementations, “n_layers” is the number of hidden layers plus 1, “batchnorm_layerN” determines whether to apply batch normalization to the outputs of the Nth layer, and “dropout_layerN” and “n_units_layerN” refer to the dropout frequency applied to nodes in the Nth layer and the number of nodes in the Nth layer respectively. “activation” is the activation function applied to node outputs, “optimizer” is the optimizer employed by the model, “lr” is the learning rate, and “weight_decay” is the L2 regularization penalty.

*LightGBM*[64] *search space*.

num_iterations: Integer between 100 and 1000. Step size of 50.

num_leaves: Integer between 2 and 3002. Step size of 20.
 learning_rate: Floating point number between .01 and .3.
 min_child_weight: Floating point number between .01 and 20.
 min_data_in_leaf: Integer between 5 and 100.
 max_depth: Integer between -1 and 20.
 colsample_bytree: Floating point number .4 and 1.
 Subsample: Floating point number .4 and 1.
 reg_lambda: Integer between 0 and 100.
 reg_alpha: Integer point number between 0 and 100.
 is_unbalance: Boolean.

*RandomForest search space.*

n_estimators: Integer between 100 and 1000. Step size of 100.
 max_features: One of: “auto”, “sqrt”, “log2”.
 max_depth: Integer between 1 and 200 or “None”.
 min_samples_split: Floating point number between .00001 and 1.
 min_samples_leaf: Integer between 1 and 20.
 booststrap: Boolean.

*XGBoost search space.*

learning_rate: Floating point number between .001 and .401. Step size of .001.
 min_child_weight: Integer between 0 and 200.
 max_depth: Integer between 1 and 200.
 colsample_bytree: Floating point number between .4 and 1. Step size of .1 .
 colsample_bylevel: Floating point number between .4 and 1. Step size of .1 .
 max_delta_step: Integer between 0 and 10.
 gamma: Floating point number between 0 and 100.
 reg_lambda: Integer between 0 and 100.
 reg_alpha: Integer between 0 and 100.
 subsample: Floating point number between .4 and 1. Step size of .1.

*Neural Network search space.* n_layers: Integer between 2 and 5.
 batchnorm_layerN: One of: “True”, “False”.
 n_units_layerN: Integer between 128 and 2,056.
 dropout_layerN: Floating point number between 0 and .9. Step size of .01.
 activation: One of: “LeakyReLU”, “ReLU”, “Sigmoid”, “Tanh”.
 optimizer: One of: “Adam”, “RMSprop”, “SGD”.
 lr: Floating point number between .00001 and 1 sampled from the log-uniform distribution.
 weight_decay: One of: .00001, .0001, .001, .01, .1, 0.
 batch_size: Integer between 256 and 1,024.
 do_classweight: Boolean indicating whether to weight samples inversely proportional to class frequencies.

**Supplementary Figures**

**Fig. S1**

**
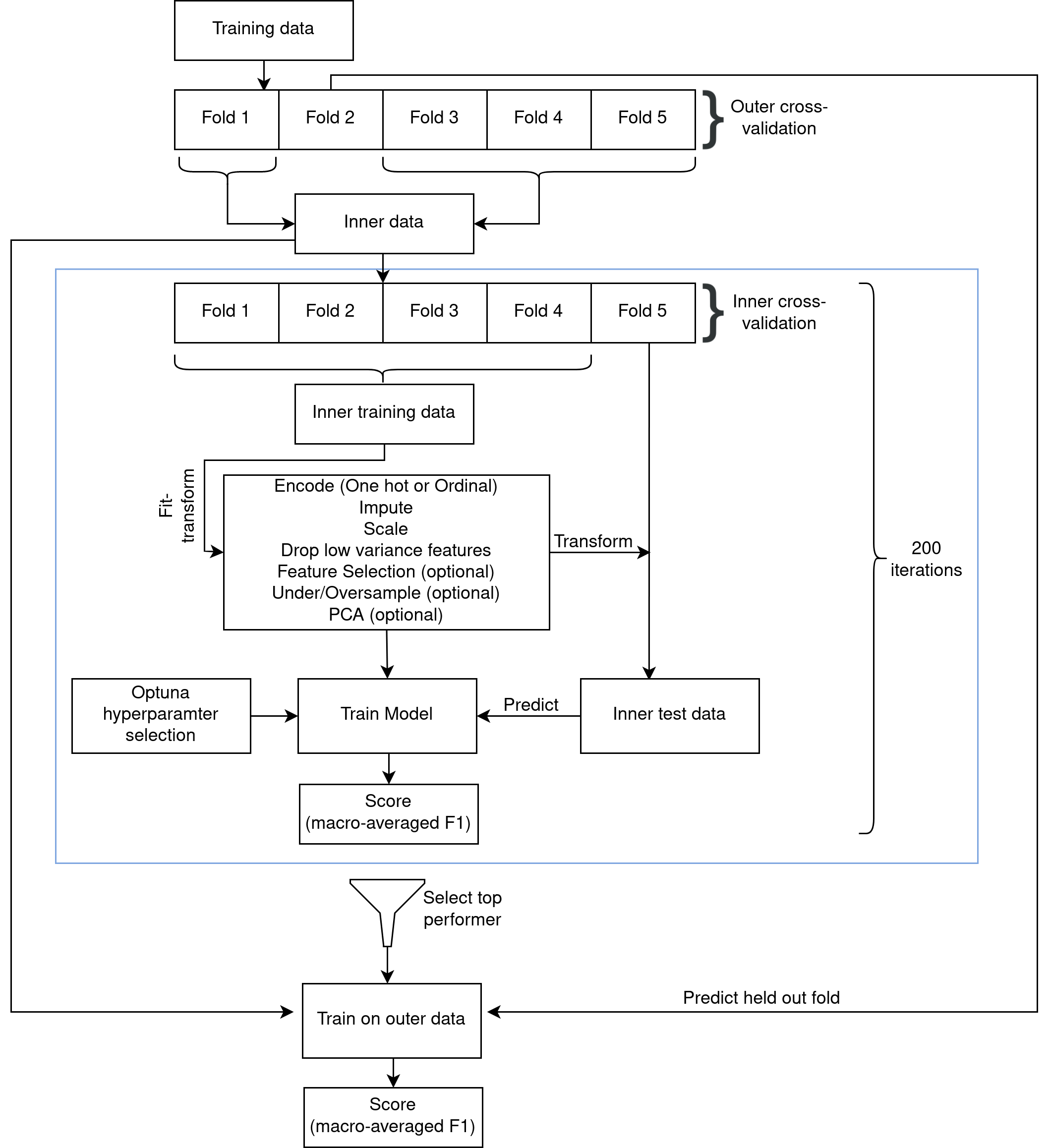
**

**Fig. S1**: Nested cross-validation strategy employed for model selection. Illustration of the nested cross-validation strategy employed for model selection in which fold two is held out for an iteration of the outer validation loop and fold five is held out for an iteration of the inner loop. Folds are stratified by variant class and split such that the set of genes in each fold is disjoint. Preprocessing steps and hyperparameters are tuned in the inner loop and performance is assessed by the macro-averaged F1 score in both the inner and outer loops.

**Fig. S2**


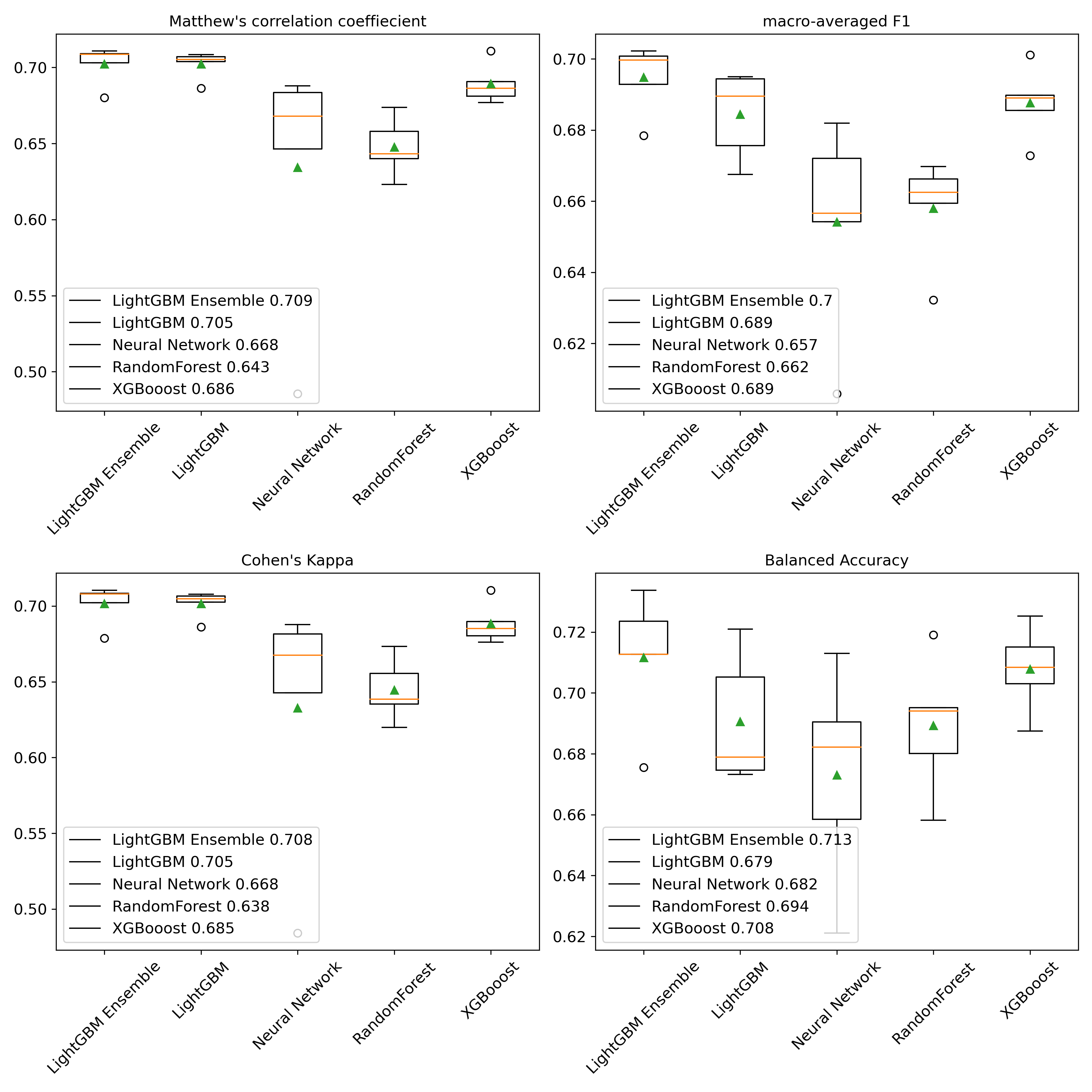


**Fig. S2**: Box and whisker plots for the comparison of tested model architecture performance on the outer folds of the nested cross-validation loop. Models are compared by respective Matthew’s Correlation Coefficient, Cohen’s kappa, macro-averaged F1, and balanced accuracy scores. Green triangles denote the mean score by model and orange horizontal lines denote the median score by model. Boxes extend from the first to the third quartiles. Whiskers extend by 1.5 times the interquartile range past the boxes. Values beyond the whisker range are denoted by circles.

**Fig. S3**

**
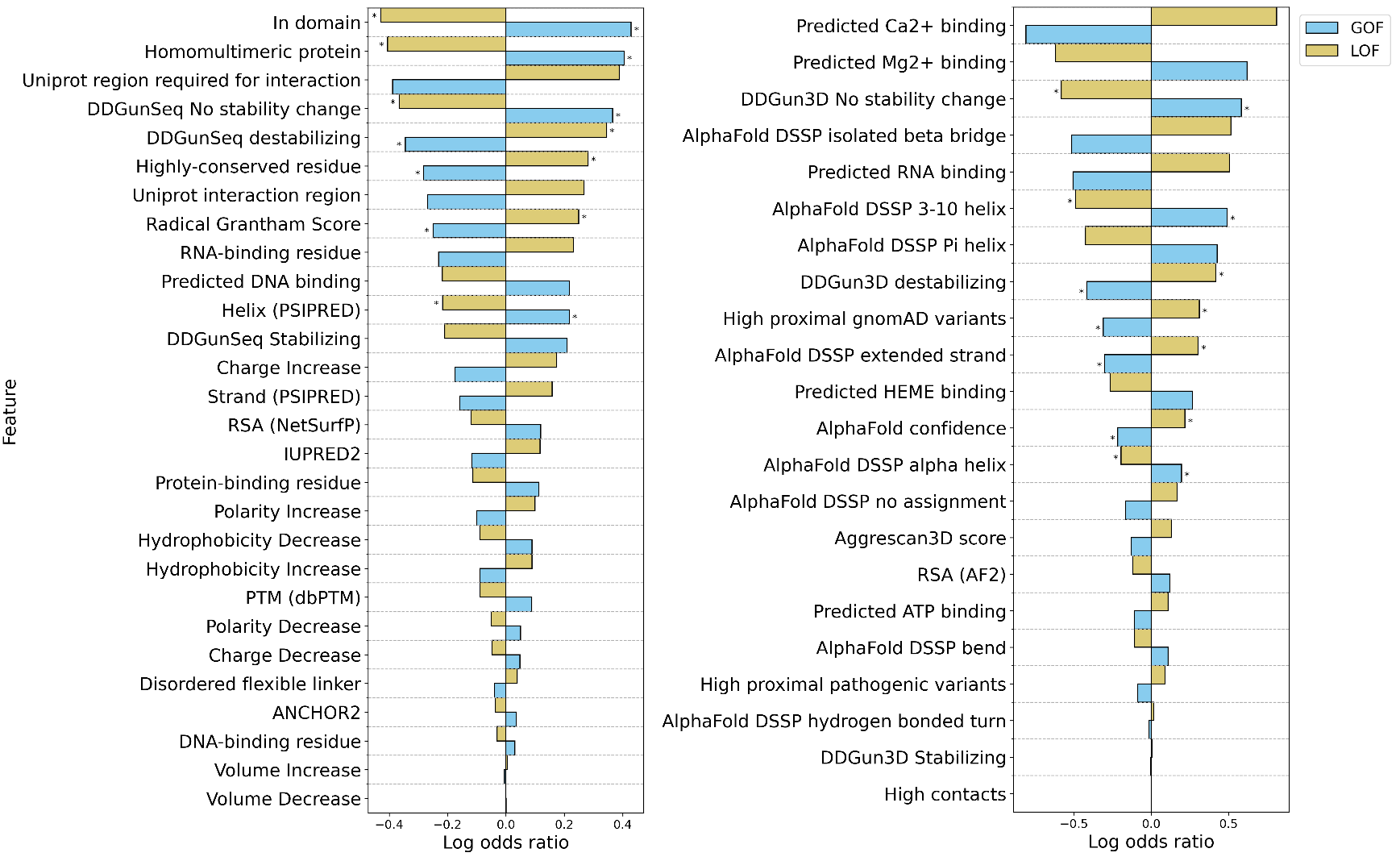
**

**Fig. S3:** Enrichments and depletions for protein structural and functional features used by the LoGoFunc model. GOF (blue) and LOF (gold) log odds ratios are displayed for each feature. Significant enrichments and depletions are denoted by asterisks. Significance was calculated with Fisher's exact test, Benjamini-Hochberg [69] corrected to allow for multiple comparisons. (Left) Features derived from protein sequences or protein interaction data. (Right) Features derived from AlphaFold2 [70] protein structures.

**Fig. S4**

**
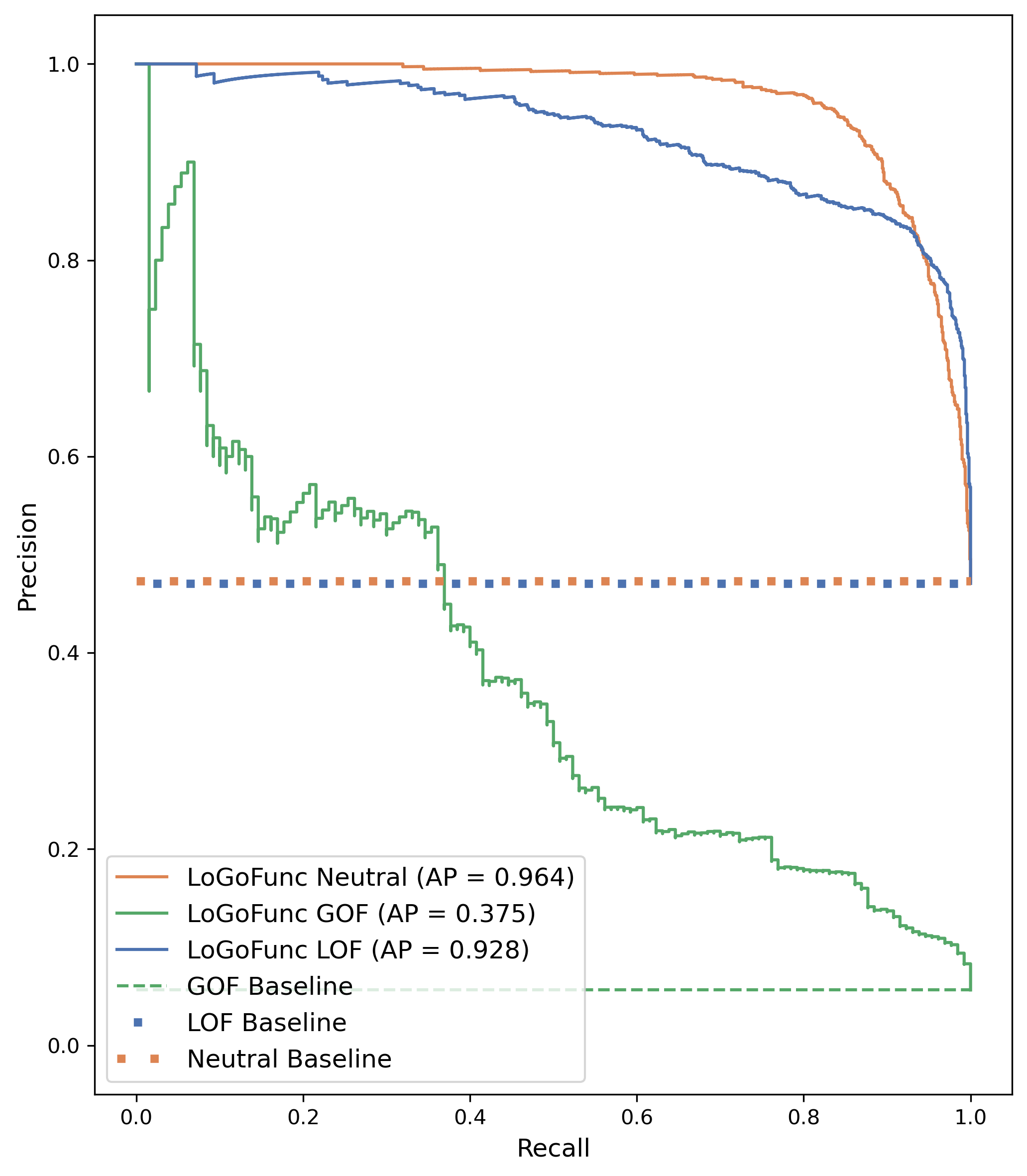
**

**Fig. S4:** Precision-recall curves by variant class for all variants in the homology-filtered test set.

**Fig. S5**


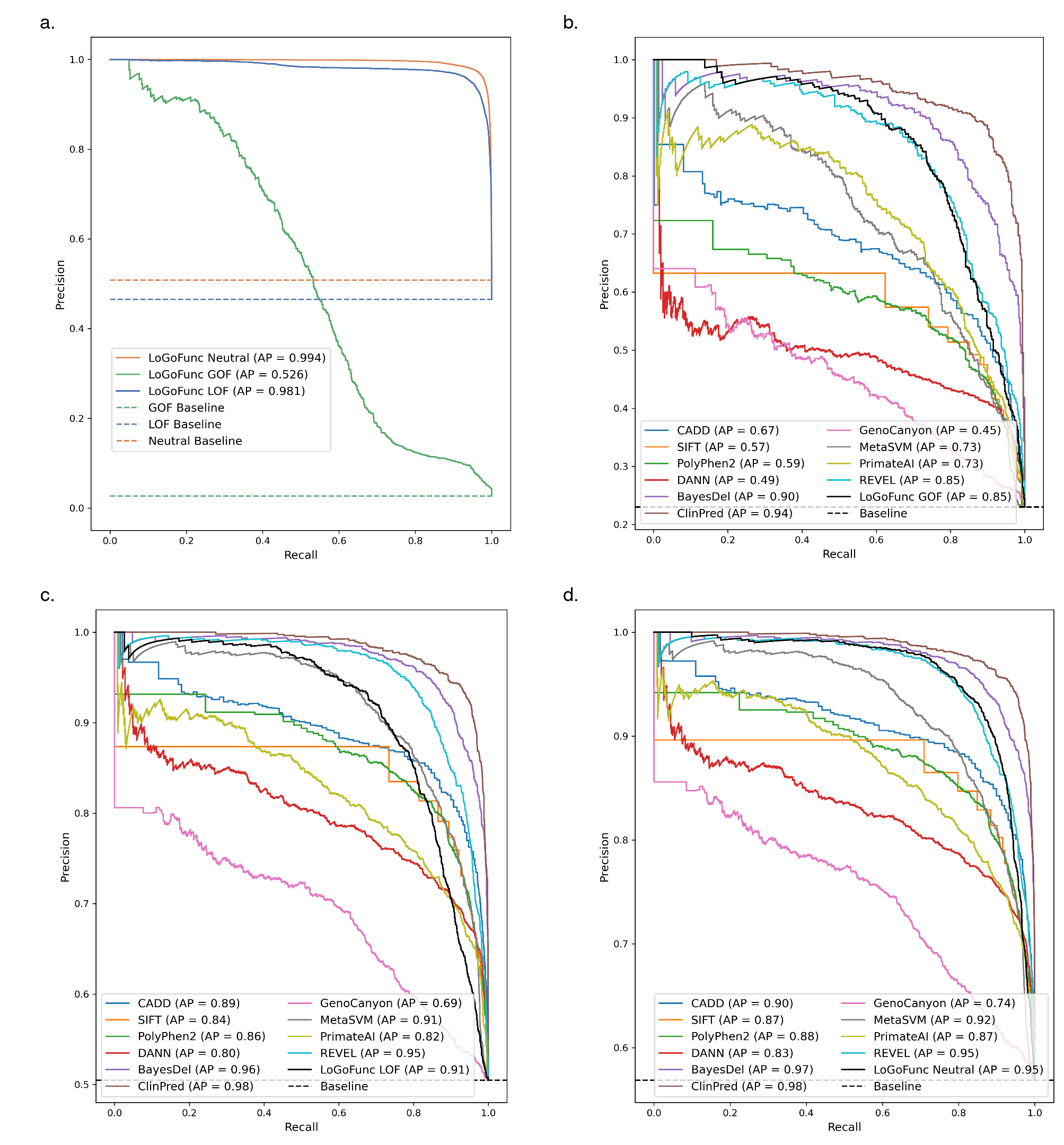


**Fig. S5**: Precision-recall curves indicating the discriminatory power of various pathogenicity prediction methods and LoGoFunc on a set of variants from ClinVar for which predictions were available from all compared tools. **a.** LoGoFunc’s performance on all testing variants (n. GOF=508, n. LOF=1,730, n. neutral=1,697). **b.** GOF (n. 508) *vs*. neutral (n. 1,697). **c**. LOF (n. 1,730) *vs.* neutral (n. 1,697). **d.** GOF (n. 508) and LOF (n. 1,730) combined *vs.* neutral (n. 1,697).

**Fig. S6**


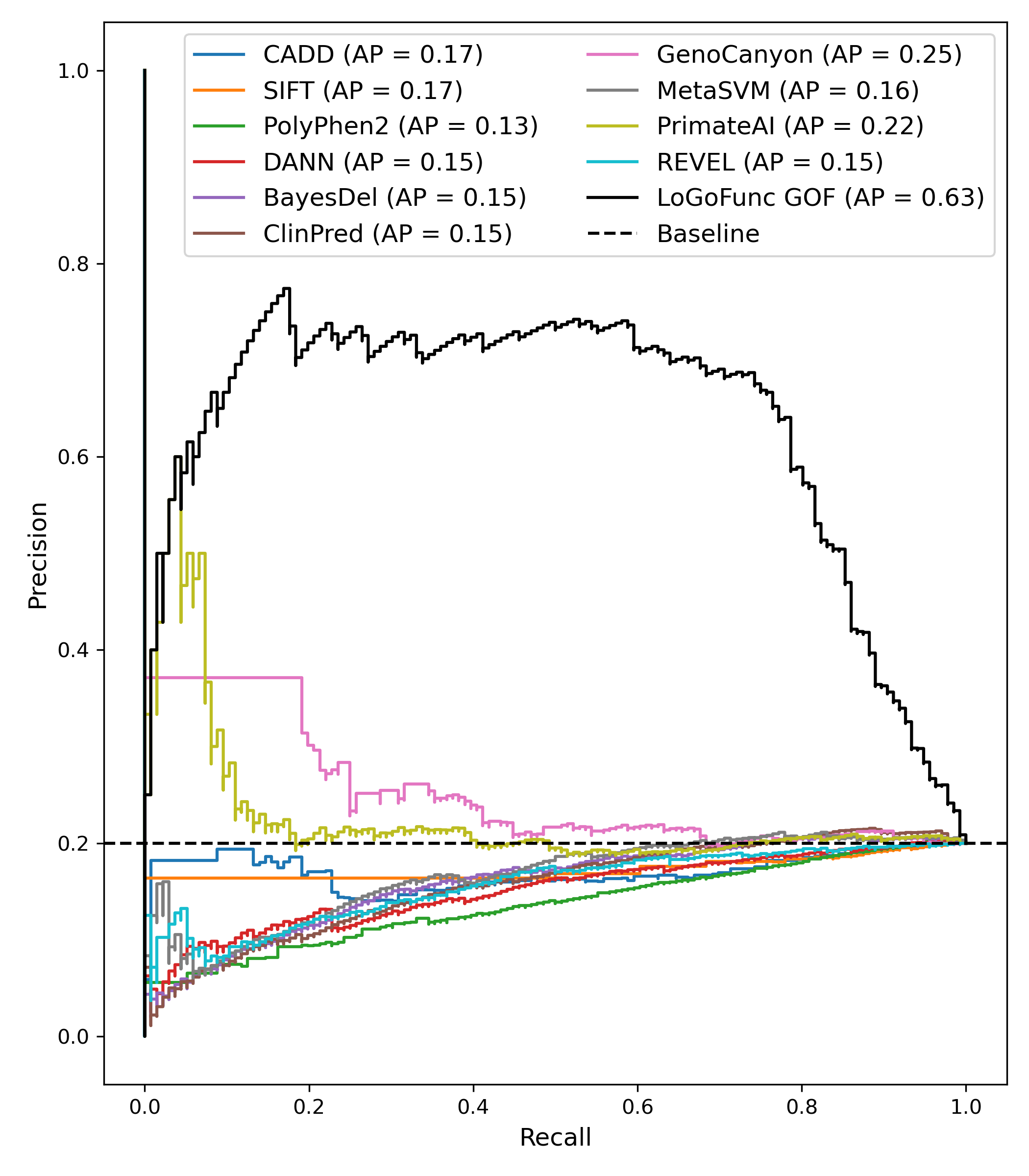
**Fig. S6**: Precision-recall curves indicating the discriminatory power of various pathogenicity prediction methods and LoGoFunc on variants from the test set for which predictions were available from all compared tools. GOF (n. 136) *vs*. LOF (n. 545).

**Fig. S7**

**
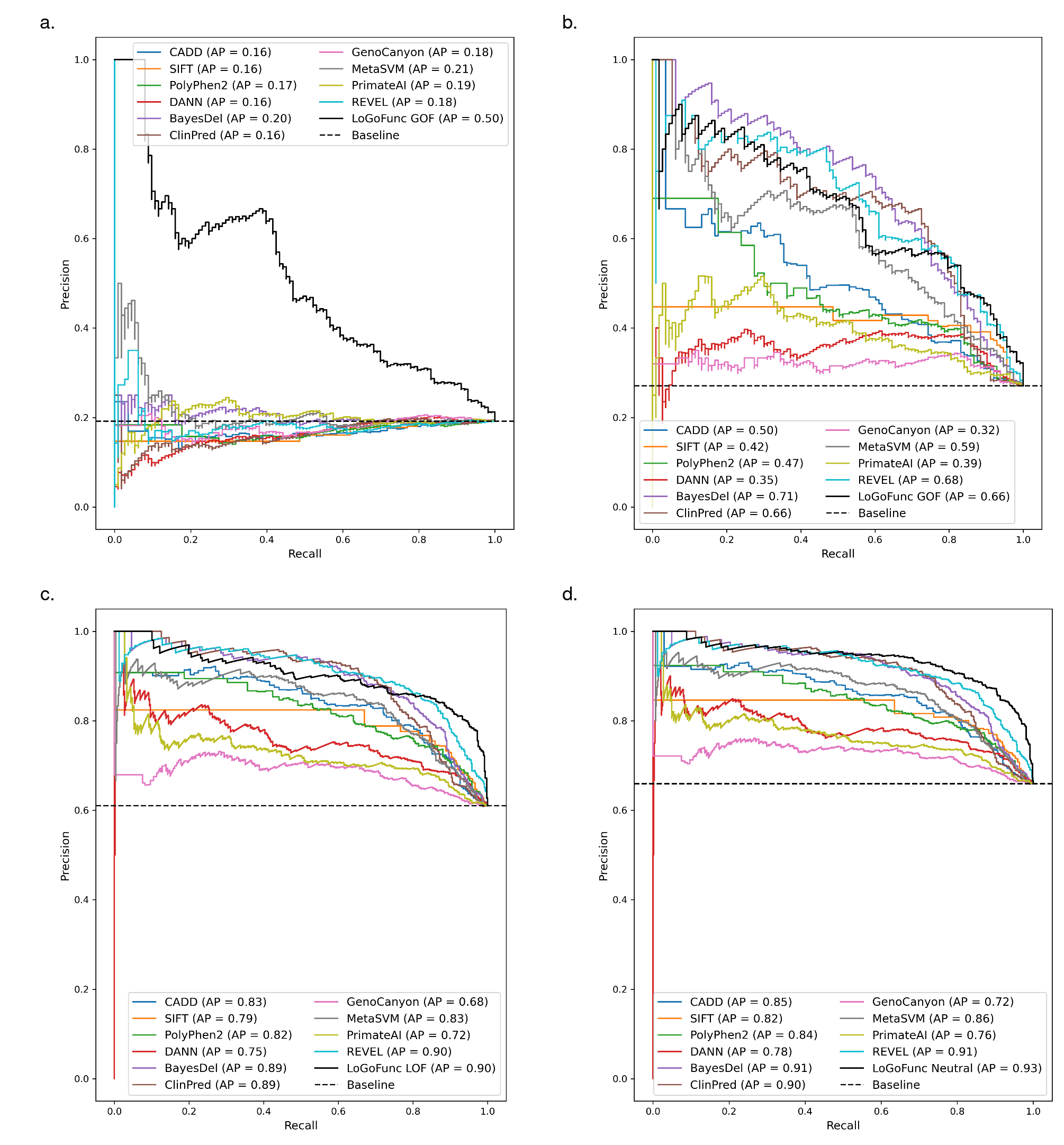
**

**Fig. S7**: Precision-recall curves comparing the discriminatory power of various pathogenicity prediction methods and LoGoFunc on a set of variants from the homology-filtered test set for which predictions were available from all compared tools. **a**. GOF (n. 113) *vs.* LOF (n. 476). **b.** GOF (n. 113) *vs*. neutral (n. 304). **c**. LOF (n. 476) *vs.* neutral (n. 304). **d.** GOF (n. 113) and LOF (n. 476) combined *vs.* neutral (n. 304).

**Fig. S8**


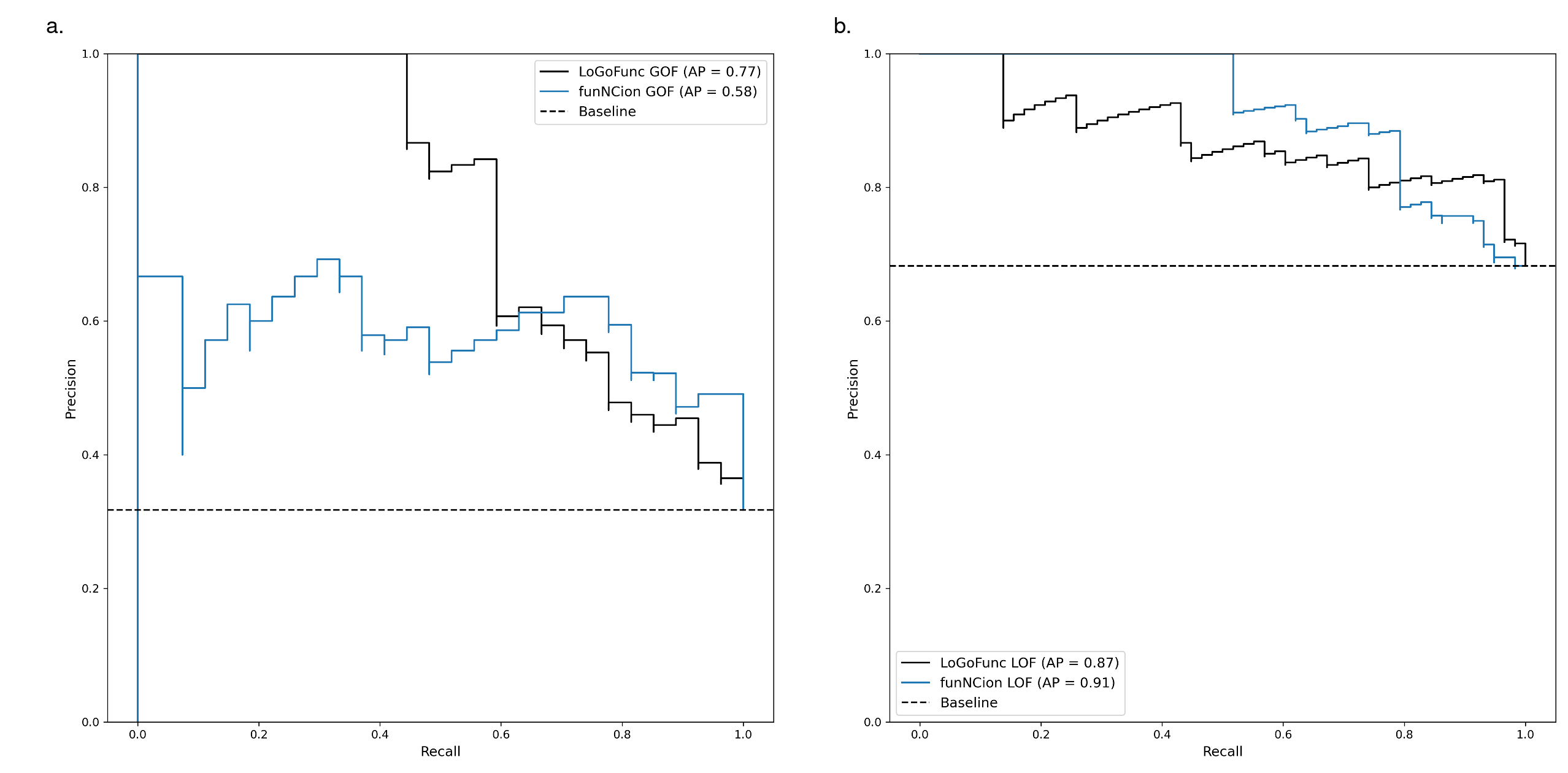
**Fig. S8:** Precision-recall curves comparing the discriminatory power of funNCion and LoGoFunc on variants from the funNCion testing dataset. **a**. Positive class GOF (n. 27) *vs.* LOF (n. 58). **b.** Positive class LOF (n. 58) *vs.* GOF (n. 27).

**Fig. S9**


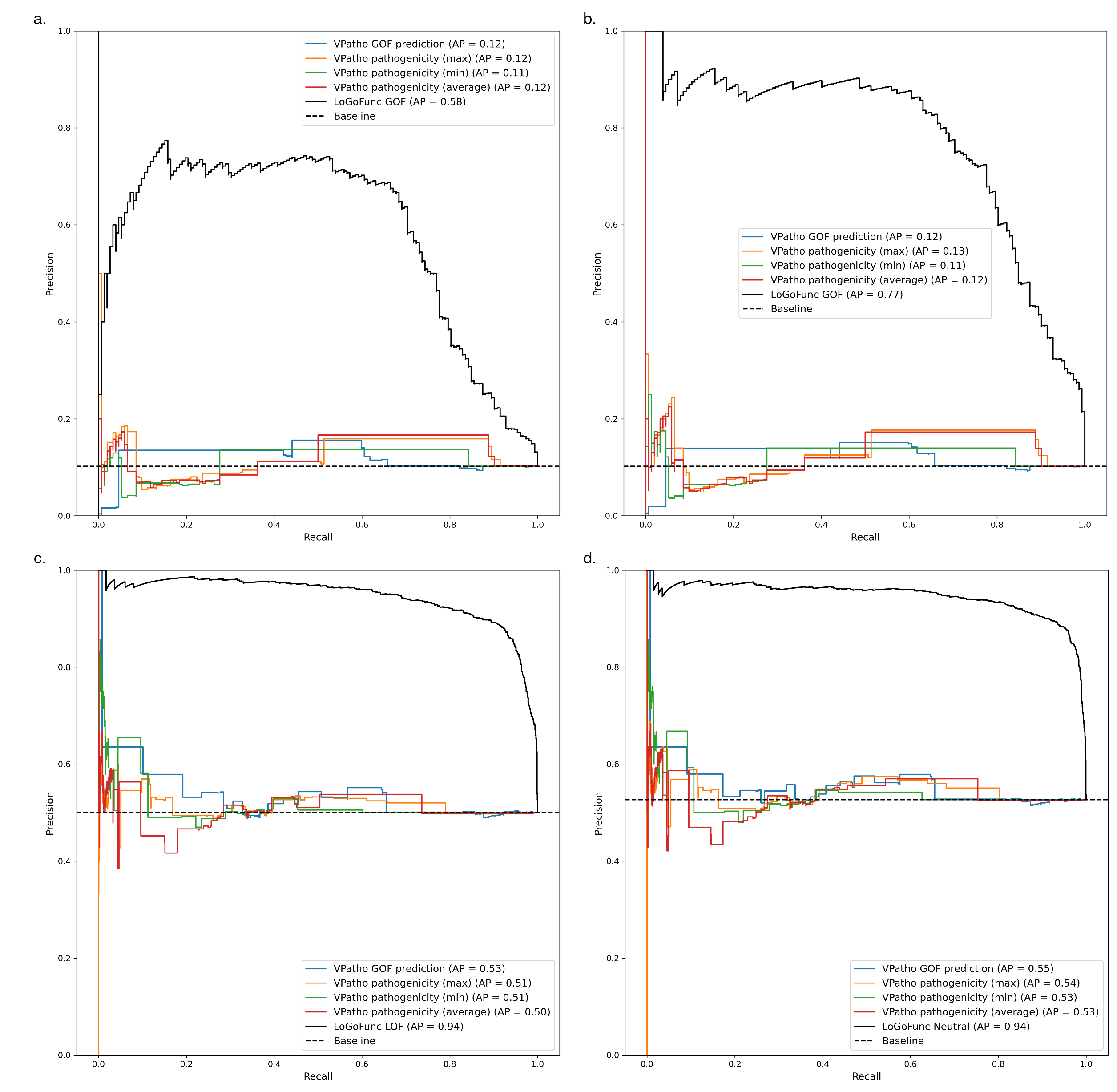
**Fig. S9:** Precision-recall curves comparing the discriminatory power of VPatho and LoGoFunc on a set of variants from the test set for which predictions were available from both tools. **a**. GOF (n. 152) *vs.* LOF (n. 1,339). **b.** GOF (n. 152) *vs*. neutral (n. 1,339). **c**. LOF (n. 1,339) *vs.* neutral (n. 1,339). **d.** GOF (n. 152) and LOF (n. 1,339) combined *vs.* neutral (n. 1,339).

**Fig. S10**


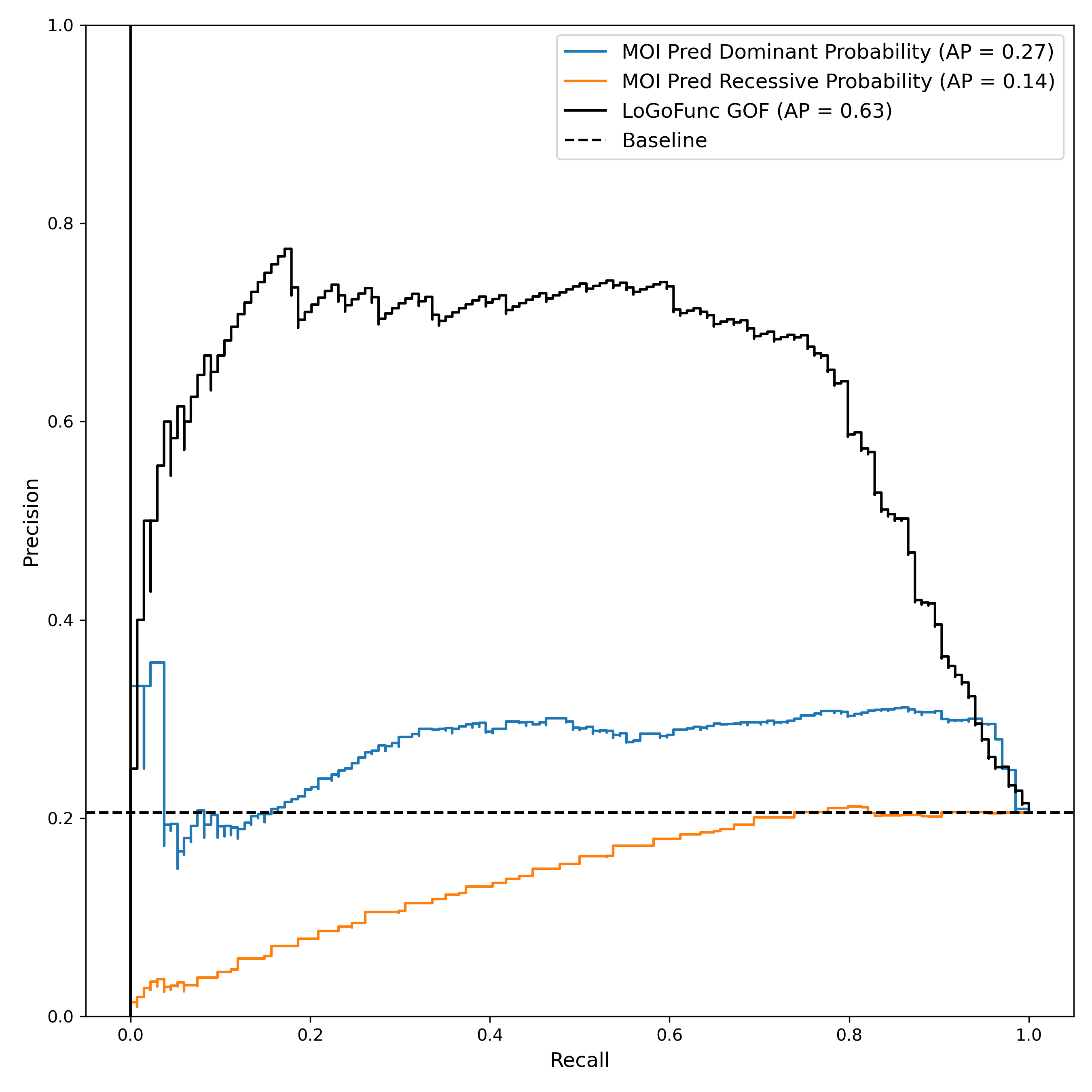
**Fig. S10:** Precision-recall curves indicating the discriminatory power of mode of inheritance predictions from MOI-pred [71] and LoGoFunc on variants from the test set for which predictions were available from each compared tool. GOF (n. 134) *vs*. LOF (n. 518).

**Fig. S11**


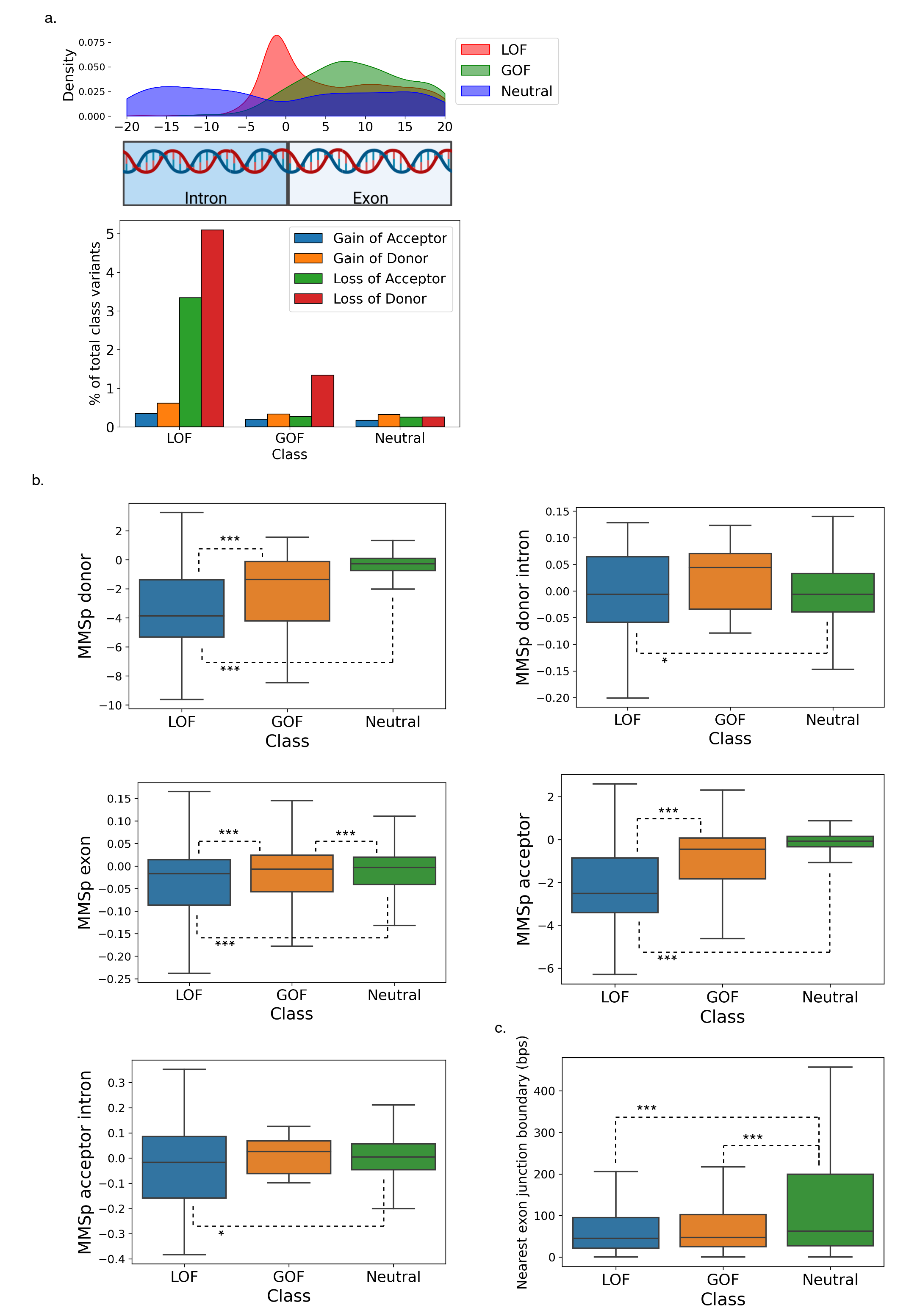
**Fig. S11: a.** (Top) Density of GOF, LOF and neutral variants within 20 base-pairs of a splice junction. (Bottom) Proportion of GOF, LOF, and neutral variants predicted to yield a gain of splice acceptor or donor or a loss of splice acceptor or donor. **b.** MMSplice [56] sub-model alternate minus reference logit percent-spliced-in predictions for variants predicted to impact splicing. **c.** Distance to the nearest exon junction boundary in nucleotides by variant class. Boxes denote quartiles, whilst whiskers extend to the limits of the distribution with outliers not shown when greater than 1.5 times the interquartile range from the low and high quartiles respectively. Created with [BioRender.com](about:blank). * *P* < .05, ** *P* < .01, *** *P* < .001

**Supplementary References**

1. Siepel A, Bejerano G, Pedersen JS, Hinrichs AS, Hou M, Rosenbloom K, et al. Evolutionarily conserved elements in vertebrate, insect, worm, and yeast genomes. Genome Res. 2005;15:1034–50.

2. Rentzsch P, Schubach M, Shendure J, Kircher M. CADD-Splice—improving genome-wide variant effect prediction using deep learning-derived splice scores. Genome Med. 2021;13:31.

3. Karczewski KJ, Francioli LC, Tiao G, Cummings BB, Alföldi J, Wang Q, et al. The mutational constraint spectrum quantified from variation in 141,456 humans. Nature. 2020;581:434–43.

4. Liu X, Li C, Mou C, Dong Y, Tu Y. dbNSFP v4: a comprehensive database of transcript-specific functional predictions and annotations for human nonsynonymous and splice-site SNVs. Genome Med. 2020;12:103.

5. McLaren W, Gil L, Hunt SE, Riat HS, Ritchie GRS, Thormann A, et al. The Ensembl Variant Effect Predictor. Genome Biol. 2016;17:122.

6. Grantham R. Amino acid difference formula to help explain protein evolution. Science. 1974;185:862–4.

7. Yampolsky LY, Stoltzfus A. Untangling the effects of codon mutation and amino acid exchangeability. Pac Symp Biocomput Pac Symp Biocomput. 2005;433–44.

8. Dayhoff MO, Schwartz RM. Chapter 22: A model of evolutionary change in proteins. Atlas Protein Seq Struct. 1978.

9. Miyazawa S, Jernigan RL. Estimation of effective interresidue contact energies from protein crystal structures: quasi-chemical approximation. Macromolecules. 1985;18:534–52.

10. Venkatarajan MS, Braun W. New quantitative descriptors of amino acids based on multidimensional scaling of a large number of physical–chemical properties. Mol Model Annu. 2001;7:445–53.

11. Wong WC, Kim D, Carter H, Diekhans M, Ryan MC, Karchin R. CHASM and SNVBox: toolkit for detecting biologically important single nucleotide mutations in cancer. Bioinformatics. 2011;27:2147–8.

12. Davydov EV, Goode DL, Sirota M, Cooper GM, Sidow A, Batzoglou S. Identifying a High Fraction of the Human Genome to be under Selective Constraint Using GERP++. PLoS Comput Biol. 2010;6:e1001025.

13. Garber M, Guttman M, Clamp M, Zody MC, Friedman N, Xie X. Identifying novel constrained elements by exploiting biased substitution patterns. Bioinformatics. 2009;25:i54–62.

14. Sunyaev SR, Eisenhaber F, Rodchenkov IV, Eisenhaber B, Tumanyan VG, Kuznetsov EN. PSIC: profile extraction from sequence alignments with position-specific counts of independent observations. Protein Eng. 1999;12:387–94.

15. Adzhubei IA, Schmidt S, Peshkin L, Ramensky VE, Gerasimova A, Bork P, et al. A method and server for predicting damaging missense mutations. Nat Methods. 2010;7:248–9.

16. Cooper GM, Stone EA, Asimenos G, NISC Comparative Sequencing Program, Green ED, Batzoglou S, et al. Distribution and intensity of constraint in mammalian genomic sequence. Genome Res. 2005;15:901–13.

17. Steinegger M, Söding J. MMseqs2 enables sensitive protein sequence searching for the analysis of massive data sets. Nat Biotechnol. 2017;35:1026–8.

18. Zhao B, Katuwawala A, Oldfield CJ, Dunker AK, Faraggi E, Gsponer J, et al. DescribePROT: database of amino acid-level protein structure and function predictions. Nucleic Acids Res. 2021;49:D298–308.

19. Fairley S, Lowy-Gallego E, Perry E, Flicek P. The International Genome Sample Resource (IGSR) collection of open human genomic variation resources. Nucleic Acids Res. 2020;48:D941–7.

20. Moayyeri A, Hammond CJ, Hart DJ, Spector TD. The UK Adult Twin Registry (TwinsUK Resource). Twin Res Hum Genet Off J Int Soc Twin Stud. 2013;16:144–9.

21. Forbes S, Clements J, Dawson E, Bamford S, Webb T, Dogan A, et al. COSMIC 2005. Br J Cancer. 2006;94:318–22.

22. Schmidt CW. HapMap: Building a Database with Blocks. Environ Health Perspect. 2003;111:a16–a16.

23. UniProt Consortium. UniProt: the universal protein knowledgebase in 2021. Nucleic Acids Res. 2021;49:D480–9.

24. Huang H, Arighi CN, Ross KE, Ren J, Li G, Chen S-C, et al. iPTMnet: an integrated resource for protein post-translational modification network discovery. Nucleic Acids Res. 2018;46:D542–50.

25. Li Z, Li S, Luo M, Jhong J-H, Li W, Yao L, et al. dbPTM in 2022: an updated database for exploring regulatory networks and functional associations of protein post-translational modifications. Nucleic Acids Res. 2022;50:D471–9.

26. Petersen B, Petersen TN, Andersen P, Nielsen M, Lundegaard C. A generic method for assignment of reliability scores applied to solvent accessibility predictions. BMC Struct Biol. 2009;9:51.

27. Mészáros B, Erdos G, Dosztányi Z. IUPred2A: context-dependent prediction of protein disorder as a function of redox state and protein binding. Nucleic Acids Res. 2018;46:W329–37.

28. Mistry J, Chuguransky S, Williams L, Qureshi M, Salazar GA, Sonnhammer ELL, et al. Pfam: The protein families database in 2021. Nucleic Acids Res. 2021;49:D412–9.

29. Blum M, Chang H-Y, Chuguransky S, Grego T, Kandasaamy S, Mitchell A, et al. The InterPro protein families and domains database: 20 years on. Nucleic Acids Res. 2021;49:D344–54.

30. Howe KL, Achuthan P, Allen J, Allen J, Alvarez-Jarreta J, Amode MR, et al. Ensembl 2021. Nucleic Acids Res. 2021;49:D884–91.

31. Vaser R, Adusumalli S, Leng SN, Sikic M, Ng PC. SIFT missense predictions for genomes. Nat Protoc. 2016;11:1–9.

32. Feng B-J. PERCH: A Unified Framework for Disease Gene Prioritization. Hum Mutat. 2017;38:243–51.

33. Alirezaie N, Kernohan KD, Hartley T, Majewski J, Hocking TD. ClinPred: Prediction Tool to Identify Disease-Relevant Nonsynonymous Single-Nucleotide Variants. Am J Hum Genet. 2018;103:474–83.

34. Quang D, Chen Y, Xie X. DANN: a deep learning approach for annotating the pathogenicity of genetic variants. Bioinforma Oxf Engl. 2015;31:761–3.

35. Raimondi D, Tanyalcin I, Ferté J, Gazzo A, Orlando G, Lenaerts T, et al. DEOGEN2: prediction and interactive visualization of single amino acid variant deleteriousness in human proteins. Nucleic Acids Res. 2017;45:W201–6.

36. Ionita-Laza I, McCallum K, Xu B, Buxbaum JD. A spectral approach integrating functional genomic annotations for coding and noncoding variants. Nat Genet. 2016;48:214–20.

37. Shihab HA, Gough J, Cooper DN, Stenson PD, Barker GLA, Edwards KJ, et al. Predicting the functional, molecular, and phenotypic consequences of amino acid substitutions using hidden Markov models. Hum Mutat. 2013;34:57–65.

38. Huang Y-F, Gulko B, Siepel A. Fast, scalable prediction of deleterious noncoding variants from functional and population genomic data. Nat Genet. 2017;49:618–24.

39. Malhis N, Jacobson M, Jones SJM, Gsponer J. LIST-S2: taxonomy based sorting of deleterious missense mutations across species. Nucleic Acids Res. 2020;48:W154–61.

40. Chun S, Fay JC. Identification of deleterious mutations within three human genomes. Genome Res. 2009;19:1553–61.

41. Jagadeesh KA, Wenger AM, Berger MJ, Guturu H, Stenson PD, Cooper DN, et al. M-CAP eliminates a majority of variants of uncertain significance in clinical exomes at high sensitivity. Nat Genet. 2016;48:1581–6.

42. Samocha KE, Kosmicki JA, Karczewski KJ, O’Donnell-Luria AH, Pierce-Hoffman E, MacArthur DG, et al. Regional missense constraint improves variant deleteriousness prediction [Internet]. bioRxiv; 2017 [cited 2022 Mar 31]. p. 148353. Available from: https://www.biorxiv.org/content/10.1101/148353v1

43. Qi H, Zhang H, Zhao Y, Chen C, Long JJ, Chung WK, et al. MVP predicts the pathogenicity of missense variants by deep learning. Nat Commun. 2021;12:510.

44. Li B, Krishnan VG, Mort ME, Xin F, Kamati KK, Cooper DN, et al. Automated inference of molecular mechanisms of disease from amino acid substitutions. Bioinforma Oxf Engl. 2009;25:2744–50.

45. Reva B, Antipin Y, Sander C. Predicting the functional impact of protein mutations: application to cancer genomics. Nucleic Acids Res. 2011;39:e118.

46. Schwarz JM, Cooper DN, Schuelke M, Seelow D. MutationTaster2: mutation prediction for the deep-sequencing age. Nat Methods. 2014;11:361–2.

47. Choi Y, Sims GE, Murphy S, Miller JR, Chan AP. Predicting the functional effect of amino acid substitutions and indels. PloS One. 2012;7:e46688.

48. Sundaram L, Gao H, Padigepati SR, McRae JF, Li Y, Kosmicki JA, et al. Predicting the clinical impact of human mutation with deep neural networks. Nat Genet. 2018;50:1161–70.

49. Carter H, Douville C, Stenson PD, Cooper DN, Karchin R. Identifying Mendelian disease genes with the variant effect scoring tool. BMC Genomics. 2013;14 Suppl 3:S3.

50. Itan Y, Shang L, Boisson B, Ciancanelli MJ, Markle JG, Martinez-Barricarte R, et al. The mutation significance cutoff: gene-level thresholds for variant predictions. Nat Methods. 2016;13:109–10.

51. Gulko B, Hubisz MJ, Gronau I, Siepel A. A method for calculating probabilities of fitness consequences for point mutations across the human genome. Nat Genet. 2015;47:276–83.

52. Kircher M, Witten DM, Jain P, O’Roak BJ, Cooper GM, Shendure J. A general framework for estimating the relative pathogenicity of human genetic variants. Nat Genet. 2014;46:310–5.

53. Ng PC, Henikoff S. SIFT: predicting amino acid changes that affect protein function. Nucleic Acids Res. 2003;31:3812–4.

54. González-Pérez A, López-Bigas N. Improving the Assessment of the Outcome of Nonsynonymous SNVs with a Consensus Deleteriousness Score, Condel. Am J Hum Genet. 2011;88:440–9.

55. Jaganathan K, Kyriazopoulou Panagiotopoulou S, McRae JF, Darbandi SF, Knowles D, Li YI, et al. Predicting Splicing from Primary Sequence with Deep Learning. Cell. 2019;176:535-548.e24.

56. Cheng J, Nguyen TYD, Cygan KJ, Çelik MH, Fairbrother WG, Avsec žiga, et al. MMSplice: modular modeling improves the predictions of genetic variant effects on splicing. Genome Biol. 2019;20:48.

57. Jian X, Boerwinkle E, Liu X. In silico prediction of splice-altering single nucleotide variants in the human genome. Nucleic Acids Res. 2014;42:13534–44.

58. Shamsani J, Kazakoff SH, Armean IM, McLaren W, Parsons MT, Thompson BA, et al. A plugin for the Ensembl Variant Effect Predictor that uses MaxEntScan to predict variant spliceogenicity. Bioinformatics. 2019;35:2315–7.

59. ENCODE Project Consortium. An integrated encyclopedia of DNA elements in the human genome. Nature. 2012;489:57–74.

60. Szklarczyk D, Gable AL, Lyon D, Junge A, Wyder S, Huerta-Cepas J, et al. STRING v11: protein–protein association networks with increased coverage, supporting functional discovery in genome-wide experimental datasets. Nucleic Acids Res. 2019;47:D607–13.

61. Grover A, Leskovec J. node2vec: Scalable Feature Learning for Networks. ArXiv160700653 Cs Stat [Internet]. 2016 [cited 2022 Mar 30]; Available from: http://arxiv.org/abs/1607.00653

62. Kapustin Y, Chan E, Sarkar R, Wong F, Vorechovsky I, Winston RM, et al. Cryptic splice sites and split genes. Nucleic Acids Res. 2011;39:5837–44.

63. Akiba T, Sano S, Yanase T, Ohta T, Koyama M. Optuna: A Next-generation Hyperparameter Optimization Framework. ArXiv190710902 Cs Stat [Internet]. 2019 [cited 2022 Mar 30]; Available from: http://arxiv.org/abs/1907.10902

64. Ke G, Meng Q, Finley T, Wang T, Chen W, Ma W, et al. LightGBM: a highly efficient gradient boosting decision tree. Proc 31st Int Conf Neural Inf Process Syst. Red Hook, NY, USA: Curran Associates Inc.; 2017. p. 3149–57.

65. Pedregosa F, Varoquaux G, Gramfort A, Michel V, Thirion B, Grisel O, et al. Scikit-learn: Machine Learning in Python. ArXiv12010490 Cs [Internet]. 2018 [cited 2022 Mar 30]; Available from: http://arxiv.org/abs/1201.0490

66. Chen T, Guestrin C. XGBoost: A Scalable Tree Boosting System. Proc 22nd ACM SIGKDD Int Conf Knowl Discov Data Min. 2016;785–94.

67. Breiman L. Random Forests. Mach Learn. 2001;45:5–32.

68. Paszke A, Gross S, Massa F, Lerer A, Bradbury J, Chanan G, et al. PyTorch: An Imperative Style, High-Performance Deep Learning Library. ArXiv191201703 Cs Stat [Internet]. 2019 [cited 2022 Mar 31]; Available from: http://arxiv.org/abs/1912.01703

69. Benjamini Y, Hochberg Y. Controlling the False Discovery Rate: A Practical and Powerful Approach to Multiple Testing. J R Stat Soc Ser B Methodol. 1995;57:289–300.

70. Jumper J, Evans R, Pritzel A, Green T, Figurnov M, Ronneberger O, et al. Highly accurate protein structure prediction with AlphaFold. Nature. 2021;596:583–9.

71. Petrazzini BO, Balick DJ, Forrest IS, Cho J, Rocheleau G, Jordan DM, et al. Prediction of recessive inheritance for missense variants in human disease [Internet]. medRxiv; 2021 [cited 2022 Mar 30]. p. 2021.10.25.21265472. Available from: https://www.medrxiv.org/content/10.1101/2021.10.25.21265472v1
